# SIRT1: The first key to unlocking the mystery of cardiovascular diseases

**DOI:** 10.3389/fphar.2025.1668718

**Published:** 2026-01-06

**Authors:** Khuzin Dinislam, Muneer Ahmed Khoso, Valeriy A. Kataev, Svetlana Meshcheryakova, Heng Liu, Ling Liu, Madi Guo, Song Wang, Han Lou, Yong Zhang, Xin Liu

**Affiliations:** 1 State Key Laboratory of Frigid Zone Cardiovascular Diseases (SKLFZCD), Department of Pharmacology, College of Pharmacy, and Department of Cardiology, the Second Affiliated Hospital, Harbin Medical Uni-versity, Harbin, China; 2 State Key Laboratory-Province Key Laboratories of Biomedicine-Pharmaceutics of China, and Key Laboratory of Cardiovascular Research, Ministry of Education, College of Pharmacy, Harbin, China; 3 Research Unit of Noninfectious Chronic Diseases in Frigid Zone (2019RU070), Chinese Academy of Medical Sciences, Harbin, China; 4 Bashkir State Medical University, Ministry of Health of Russia, Ufa, Russia

**Keywords:** SIRT1, cardiovascular diseases, endothelial dysfunction, oxidative stress, therapeutic targets

## Abstract

Cardiovascular disease, (CVD) the leading cause of death worldwide, encompasses various heart and vascular disorders that significantly elevate morbidity and mortality rates. Sirtuin 1 (SIRT1), a NAD^+^ dependent deacetylase, plays a key role in cardiovascular health and pathology by regulating fundamental processes such as endothelial function, oxidative stress, inflammation, senescence, metabolism, cardiac hypertrophy, and heart failure. Through these mechanisms, SIRT1 emerges as critical factor in the pathophysiology of CVDs, including heart failure, atherosclerosis, hypertension, and myocardial infarction. SIRT1 modulates several cellular pathways to regulate complex cellular processes associated with cardiovascular disorders. This review summarizes recent findings regarding the physiological and pathological roles of SIRT1 related to heart diseases and explores the therapeutic potential of targeting SIRT1 and sirtuin family members for CVD treatment.

## Graphical Abstract

**Figure:**
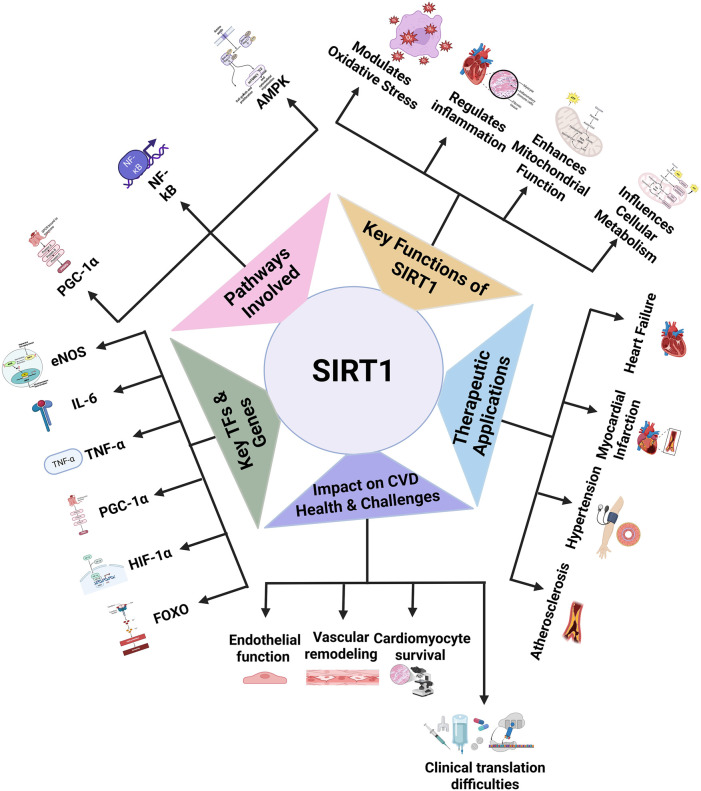

## 1 Introduction

 Cardiovascular disease is the primary cause of mortality globally, with low- and middle-income countries accounting for more than 80% of CVD-related deaths. Worldwide, the prevalence of CVD-related fatalities rose by 14.5% from 2006 to 2016, but age-standardized death rates attributed to CVD declined by 14.5% (Naghavi et al., 2017). It is anticipated that about 23.6 million individuals will succumb to CDVs by 2030, mostly due to heart disease and stroke (Yusuf et al., 2014; Dehghan et al., 2017). The SIRT family, the human homology of yeast Sir2, consists of seven members (SIRT1–SIRT7), each with distinct sequences and lengths in their N- as well as C-terminal domains (Finkel et al., 2009). SIRT is located in distinct subcellular organelles: SIRT 6/7 in the nucleus, SIRT3/4/5 in mitochondria, and SIRT1/2 in the cytoplasm and the nucleus. These proteins carry out important regulatory tasks at several subcellular locations. Growing evidence has shown that SIRTs are involved in a variety of physiological and pathobiological processes such as autophagy, inflammation, oxidative stress, genome integrity, and histone modification (Matsushima and Sadoshima, 2015). Functional studies have further validated that the molecular mechanisms underlying CVD remain inadequately understood; nonetheless, oxidative stress, cellular apoptosis, and mitochondrial dysfunction are linked to CVD pathogenesis. SIRTs are implicated in various diseases, and the pivotal function of the SIRT protein family in CVD is well established. SIRTs influence the generation of mitochondrial ROS by regulating mitochondrial activity and exacerbating endothelial dysfunction and hence are key drivers in atherosclerotic progression (Peng et al., 2019). SIRT1 is an essential regulator in CVDs, playing a key role in disease progression and acts as a possible molecular therapeutic target. This review highlights recent advances in understanding the role of SIRT1 in CVDs. Additionally, it summarizes the critical roles and pathways implicated in the pathogenesis of CVDs and the ongoing clinical investigation of agonists as well as the inhibitors of novel antioxidant compounds.

## 2 Structure, function, and regulation of SIRT1

**FIGURE 1:**
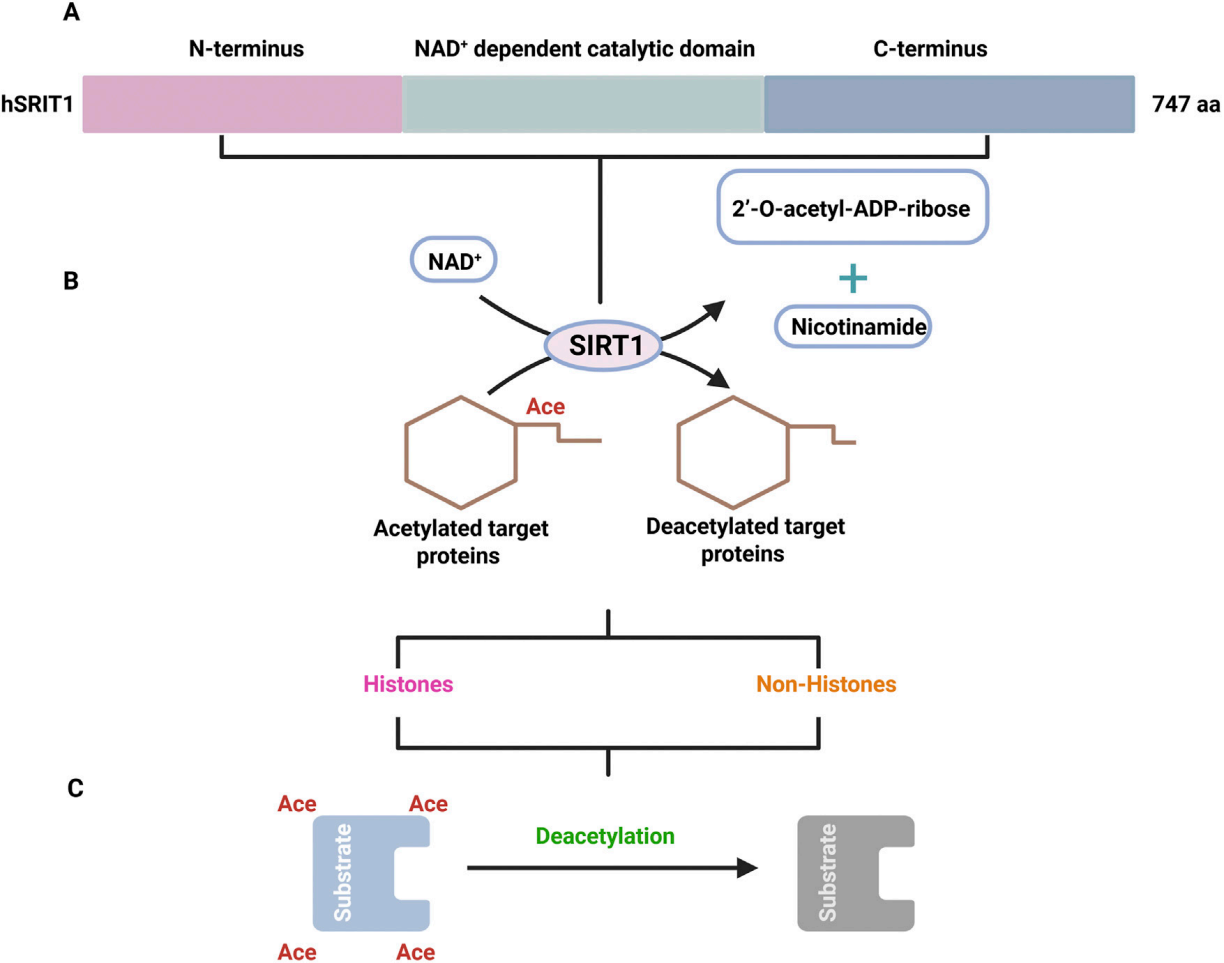
The functional and structural overview of SIRT1. **(A)** Indicating the structure of STR1 domain. **(B)** Showing that SIRT1 facilitates NAD+ dependent deacetylation of target protein, this produces deacetylated products, nicotinamide and 2’-O-acetyl-ADP-ribose. **(C)** At the end specifying that deacetylation process at the substrate within the catalytic sit.

The human SIRT1 (hrSIRT1) gene, situated on the 10q22.1 chromosome, consists of nine exons and eight introns, and it encodes a protein consisting of 747 amino acid residues, whereas the mouse SIRT1 encodes 737 of these (Figure 1A). SIRT1 is ubiquitously expressed in cross-cell types or tissue, whereas its subcellular localization varies according to cellular context, stress conditions, and interactions with other molecules. The SIRT1 protein has N-terminal, catalytic, and C-terminal domains. Its three-dimensional structure also comprises a predominant Rossmann-fold domain that is largely conserved, with a secondary domain including a zinc-binding module as well as a helical module. Catalytic activity is initiated when an acetylated residue molecule binds to NAD^+^ in the cleft between the two domains (Sauve et al., 2006; Yang et al., 2022). SIRT1 is mostly analogous to the yeast Sir2 ortholog and is a well-studied SIRT. It is mostly situated in the nucleus but may be translocated to the cytoplasm under certain circumstances. SIRT1 deacetylates acetyl-lysin residues on histone andnon-histone substrates influence several biological processes, including oxidative stress, metabolism, senescence, inflammation, and apoptosis (Ungurianu et al., 2023).

SIRT1 requires NAD^+^ for catalysis; it hydrolyzes NAD^+^ to deacetylate target proteins while concurrently moving the lysine-bound acetyl group from acetylated proteins to both 2′-O-acetyl-ADP-ribose and nicotinamide, which are produced by the 2′-OH position of ADA-ribose (Figure 1B; Imai et al., 2000; Tanner et al., 2000). SIRT1 activity and NAD^+^ levels are closely correlated by NAD^+^ dependency, and SIRT1’s many biological activities are primarily mediated via its ability to deacetylate target proteins, which may comprise both histones and non-histone proteins (Vaquero et al., 2004; Nogueiras et al., 2012). Histone proteins have a direct impact on gene transcription because of N-terminal lysin residue acetylation and the deacetylation of histones via SIRT1 (Vaquero et al., 2004; Nogueiras et al., 2012; Zhang et al., 2019). According to several studies, lysin (H1K26, H4K6, H4K12, and H3K26) and (H4K6, H4K12, and H4K16) are deacetylated via SIRT1. Moreover, the deacetylation of promoter-associated H3K9 and H4K16, by SIRT1 as well as the ensuing transcriptional repression have been extensively studied (Chen et al., 2016; Kang et al., 2018; Tatomir et al., 2020). Additionally, numerous non-histone proteins are affected by SIRT1-driven deacetylation, which is linked to pathological processes such as metabolism, inflammation, cell differentiation, and autophagy. SIRT1 directly deacetylates in order to control the TFs or co-factors, including P53, FOXO 1/3/4, HSF1, HIF-1α, NF- Κb, TIP60, and P300 (Figure 1C). Moreover, SIRT1 indirectly enhances the TFs, including MyoD (PPARα/γ) (Cheng et al., 2003; He F. et al., 2021; Huang et al., 2021; Yang et al., 2022; Ding et al., 2025).

Furthermore, the acetylated substrate is fixed at the end of the gap next to a NAD^+^ glycosylation site, with NAD binding areas located at the interface of the two domains (Alves-Fernandes and Jasiulionis, 2019). SIRT1 regulates apoptosis, cell differentiation, oxidative stress resistance, and cell cycle arrest by interacting with FOXO proteins. SIRT1 plays a critical role in the biology of aging by mediating the deacetylation of FOXO3 and FOXO4, which attenuates FOXO-induced apoptosis and increases FOXO-induced cell cycle arrest (Giannakou and Partridge, 2004). Peroxisome PGC-1α is another non-histone target of SIRT1; for nuclear receptors and other TFs, it is a nuclear transcriptional co-activator that also regulates mitochondrial biogenesis. SIRT1 facilitates mitochondrial biogenesis, increases PGC-1α activity, and protects against ischemic heart disease and brain damage by deacetylating them (Shi and Gibson, 2007; Zhou et al., 2018). Additionally, SIRT1 interacts antagonistically with NF-κB, a molecule that transfers glycolytic energy during an inflammatory response. It facilitates the resolution of inflammation through NF-κB deacetylation and by activating AMPK, PGC-1α, and PPAR-1αHowever, SIRT1 activity is reduced by NF-κB via the expression of miR-34a, IFNγ, and ROS, which causes inflammatory reactions that have been linked to a number of chronic metabolic and related diseases (Kauppinen et al., 2013). Additionally, SIRT1 improves cardiac contractility, prevents ER stress, and increases resistance to ischemia/reperfusion injury in cardiomyocytes. SIRT1 is downregulated in acute ischemia/reperfusion and is increased during physical activity, calorie restriction, and pressure overload. Consequently, a U-shaped dose-response curve was suggested as a link between SIRT1 and cardiac function, and the myocardium’s contractile ability was reduced in transgenic mice due to constitutional overexpression of SIRT1 (Ministrini et al., 2021).

SIRT1 plays a key role in protection against CVDs, metabolic syndrome, obesity, vascular endothelial function, and ischemia-reperfusion damage (Kane and Sinclair, 2018). With a wide variety of functions in cell survival, transcription control, modulation of energy metabolism, regulation of circadian rhythm, and DNA repair, mammalian sirtuins are NAD^+^ dependent deacetylases (Haigis and Sinclair, 2010; Chang and Guarente, 2014). As an essential housekeeping molecule that helps with electron transport in metabolic redox activities, NAD^+^ is a major regulator of survival pathways and cellular signalling (Hershberger et al., 2017b). NAD^+^ is a potential regulator of longevity and health since it is a primary substrate for sirtuin deacetylation, a mechanism that involves converting NAD^+^ into nicotinamide and O-acetyl-ADP-ribose in order to remove an acetyl group from target substrates (Tanner et al., 2000; Du et al., 2011). In addition to histone deacetylation, SIRT1—the most studied sirtuin—regulates TFs like p53, NFκB, FOXOs, PGC1α, and PARP1 (Vaziri et al., 2001; Yeung et al., 2004; Rodgers et al., 2005; Rajamohan et al., 2009; Mouchiroud et al., 2013). Some activators known as “sirtuin-activating compounds” (STACs) increase the activity of SIRT1 (Howitz et al., 2003). In a search for substances that increase human SIRT1 activity, resveratrol was among the first STACs discovered that prolonged yeast viability. Many STACs such as SRT11720 and SRT2104 have been developed recently, with greater potency and specificity (Hubbard and Sinclair, 2014; Bonkowski and Sinclair, 2016). Allosteric activators of the SIRT1 STACs are attached to the N-terminal STAC-binding domain and increase a substrate’s binding affinity for SIRT1 via a bend-at-the-elbow mechanism (Hubbard et al., 2013; Dai et al., 2015).

Additionally, systemic NAD production, which is regulated by SIRT1, as well as nicotinamide phosphoribosyltransferase (Nampt), is essential for controlling metabolism in mammals as well as, perhaps, aging. The NAD-dependent deacetylase and intracellular and extracellular Nampt-mediated systemic NAD production serve as catalysts to maintaining the metabolism rate in many tissues and organs. SIRT1 is a ubiquitous mediator that responds to variations in systemic NAD production by carrying out metabolic actions in a tissue-dependent manner (Imai, 2009).

### 2.1 SIRT1 in eNOS activation

SIRT1 is widely expressed in the vasculature, including the endothelial cells, perivascular adipose tissue, and smooth muscle cells (Man et al., 2019). Due to its antioxidant and anti-inflammatory characteristics, SIRT1’s function in endothelial cell biology has drawn increasing attention in research (Potente et al., 2007; Chen et al., 2013). Mice that had SIRT1 specifically deleted in endothelial cells had a reduced ability to create new capillaries in response to angiogenic stimuli (Potente et al., 2007). Elevated acetylation in heart disease is associated with deceased SIRT1 expression (Gorski et al., 2019). In an animal model (rat), the upregulation of SIRT1 has protective benefits against HF, such as decreased apoptosis and enhanced cell survival and cardiac function (Lin et al., 2020). These beneficial outcomes of SIRT1 are enhanced by eNOS activation and beneficial input processes. The synergy between SIRT1 and eNOS helps preserve endothelial function (Man et al., 2019). In a way that is dependent on NAD, SIRT1 directly binds to eNOS and deacetylates, and activates it. There is an inverse relationship between SIRT1 expression and activity and the acetylation of eNOS (Mattagajasingh et al., 2007).

SIRT1 and eNOS are linked in endothelial cells; according to current research on the basis of their co-localization in the nucleus and perinuclear cytoplasm, together they protect against endothelial cell senescence (Mattagajasingh et al., 2007; Ota et al., 2010). The eNOS is the rate-limiting enzyme for NO (a crucial neurotransmitter and signalling molecule) in the cardiovascular system. Numerous studies have verified that eNOS activation and NO generation may prevent atherosclerosis and reduce the senescence of endothelial cells (Liang et al., 2021; Niu et al., 2023). The primary constituent of caveolae, known as “caveolin-1”, binds to cytoskeletal proteins in an indirect manner, preserving caveolae invagination and preventing eNOS activation (Oliveira and Minshall, 2018). Caveolin-1 expression rises and eNOS activity falls in aged endothelium cells (Powter et al., 2015), and the eNOS-mediated NO generation may be strongly impacted by caveolin-1 delivery and binding capacity. SIRT1 regulates the amount of caveolin-1 expression and eNOS deacetylation as a deacetylase with a tight relationship to long-term extension and age delay. Research has shown that SIRT1 expression markedly declines in aged tissues, but the overexpression of SIRT1 could delay aging (Song et al., 2014; Shi et al., 2020).

However, it has found that ginsenoside (g-Rb1), a traditional Chinese medicine that is one of the main bioactive substances extracted from *Panax ginseng*, has anti-senescence properties. When g-Rb1 is administered, caveolin-1 expression was suppressed, whereas SIRT1 and eNOS mRNA and protein abundance were increased. Caveolin-1 siRNA also enhances the anti-senescence impact of g-Rb1, but siRNAs that inhibited SIRT1 and eNOS reduced it. SIRT1 siRNA inhibited G-Rb1-enhanced NO generation and lowered caveolin-1 acetylation levels. Caveolin-1 and g-Rb1 siRNAs may both increase NO production as well as lowering the acetylation level of eNOS (Zhou B. et al., 2024). Additionally, Rb1 is important for reducing oxidative stress, controlling autophagy, and preventing apoptosis. Recent studies have indicated that Rbi suppresses the SIRT1–eNOS–NO axis, hence lowering oxidative stress and inflammatory responses and thus consequently delaying the aging process in mice (Zhou et al., 2025). In mice with type 1 diabetes, icariin (flavonoid glycoside) produced from the epimedium protects the arteries by lowering inflammation linked to high-mobility group box 1 (HMGB1). When icariin was given to diabetic rats and HG-stimulated HUVECs, it increased acetylcholine-induced vasodilation in the aorta and decreased the production of pro-inflammatory cytokines such IL-8, IL-6, IL-1β, and TNF-α. Additionally, icariin activates the G-protein-coupled estrogen receptor (GPER) and SIRT1, which work together to suppress HMGB1 production and inflammation brought on by HMGB. Likewise with GPER and SIRT1 inhibitors, icariin’s impact on HMGB1 release, and HMGB1-induced inflammation, specifically, the GPER inhibitor, stopped icariin from activating SIRT1 (Wenhui et al., 2024).

## 3 Mechanisms of SIRT1 in cardiovascular diseases

**FIGURE 2:**
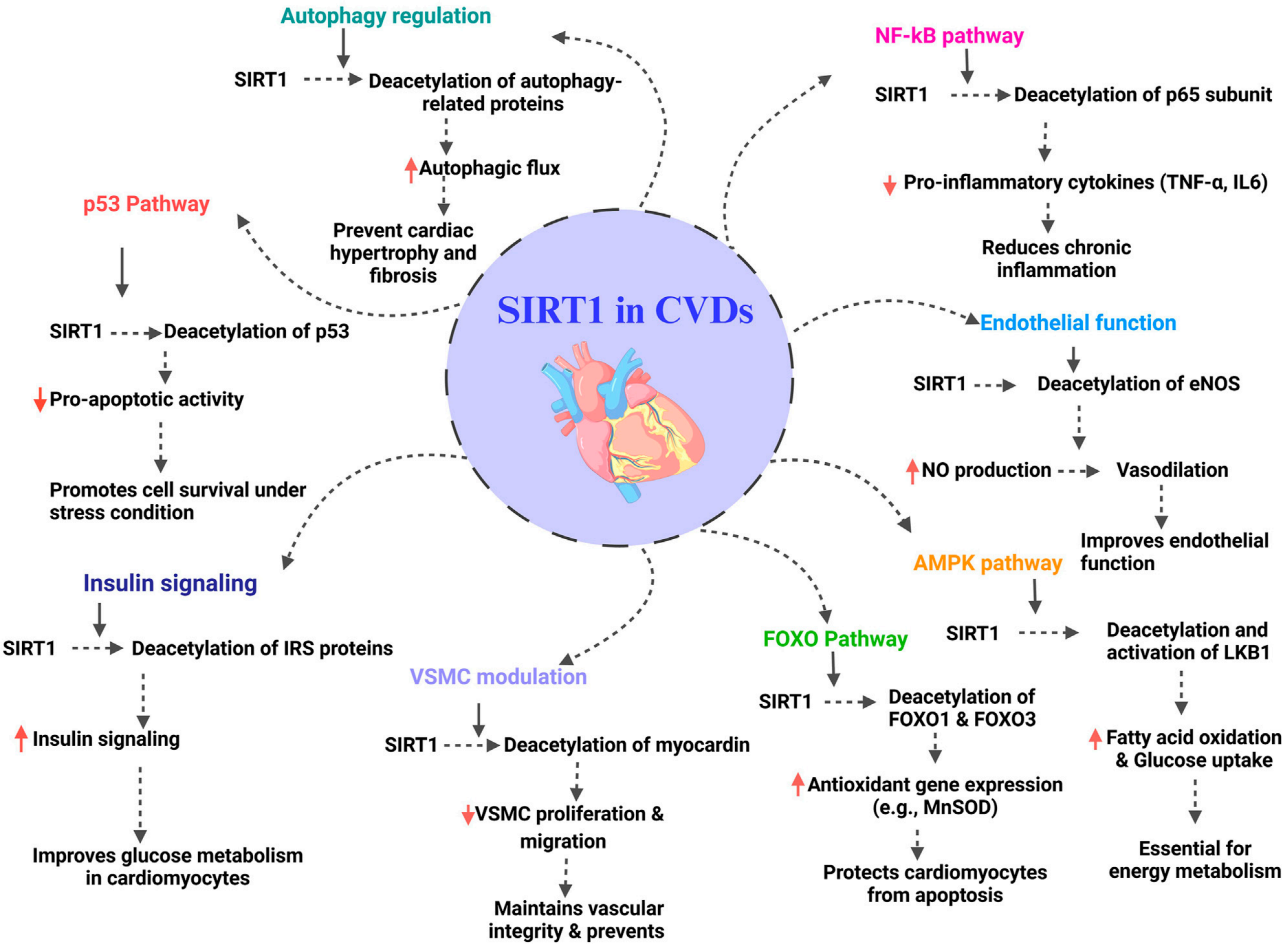
Overview of SIRT1 regulatory pathways which are involved in CVD; red arrows indicate down- and upregulation (Lee and Im, 2021).

According to the World Heart Federation, there were 20.5 million deaths due to CVD in 2021, up from 12.1 million in 1990 (Shi et al., 2025). The majority of research, both *in vitro* and *in vivo*, has effectively shown that SIRT1 is involved in both healthy and pathological cardiovascular system activities, including DNA damage, oxidative stress, apoptosis, cellular metabolism, and cellular senescence (Figure 2). Recent research has significantly advanced in the deterrence and treatment of CVDs (Table 1).

**TABLE 1:** Mechanism of SIRT1 related to CVDs.

Conditions/Stress	Model	Cell line	Functional role and targeted pathways	References
Apoptosis and ER stress	Rats	H9c2	ER stress causes cardiomyocytes to undergo apoptosis via the *IRE1α/JNK*, *ATF6/CHOP*, and *PERK/eIF2α* pathways.	Guo et al. (2015a)
Autophagy and apoptosis	Mice	H9c2	AMPK activation promotes autophagy, while IRE1α reduces hypoxia-induced apoptosis, thus protecting cardiomyocytes from hypoxic stress.	Luo et al. (2019)
Oxidative and inflammatory stress	Mice	H9c2	In cardiomyocytes, the *PI3K/MTOR* signaling pathway may reduce oxidative damage brought on by hypoxia, increase lifespan, and improve the results of interventions.	Ma et al. (2022a)
ROS	Mice	H9c2	Resveratrol has been shown to suppress the SIRT1 and mitochondrial biogenesis signaling pathways, which in turn prevent the formation of ROS in cardiomyocytes.	Li et al. (2013)
Cardiomyocyte apoptosis	Rats	H9c2	Doxorubicin-induced cardiomyopathy may benefit from treatment targeting a crucial regulator of cardiomyocyte apoptosis during doxorubicin-induced cardiac damage.	Ruan et al. (2015)
ROS	Mice	H9c2	The SIRT1 pathway-mediated antioxidative effects of H2S in H9c2 cardiomyocytes under oxidative stress.	Wu et al. (2015)
Apoptosis	Rats	H9c2	Curcumin has been shown to enhance apoptosis in diabetic rats and help cure diabetic cardiomyopathy by modifying the *SIRT1-Foxo1* and *PI3K-Akt* pathways.	Ren et al. (2020)
Ischemia reperfusion (IR) injury	Mice	H9c2	SIRT1 activation in IR tissues caused by Resv activates a protective mechanism, indicating possible therapeutic targets for the treatment of IR-induced cardiac dysfunction.	Becatti et al. (2012)
Ischemia reperfusion (IR) injury	Mice	H9c2	AMPK agonist can restore the heart’s tolerance to ischemic insults in aged and icSIRT1 KO hearts.	Wang et al. (2018)
Ischemia reperfusion (IR) injury	Mice	H9c2	Myocardial infarction treatment may target the *SIRT1/UCP-2* axis, suggesting the importance of this pathway in CVDs.	Deng et al. (2017)
Oxidative and ER stress	Mice	H9c2	It was discovered that DEX prevented the oxidative stress and ER stress-dependent apoptosis of cardiomyocytes brought on by H/R damage.	Zhang et al. (2021d)
ER stress	Rats	H9c2	SFN is proposed to protect cardiomyocytes against hypoxia/reoxygenation damage *in vitro* via activating the SIRT1 pathway and reducing ER stress-induced apoptosis.	Li et al. (2016b)
ER stress	Mice	H9c2	The deacetylation of *eIF2α* at lysine K143 by SIRT1 is a unique method for protecting cardiac cells from ER stress, indicating its activation as a prospective therapeutic strategy.	Prola et al. (2017)
Aging	Rats	H9c2	Low SIRT1 expression, linked to aging, may significantly influence the activation of proapoptotic molecules and the downregulation of antioxidants via the oxidative stress, *FoxO1*, and *p53* pathways.	Lu et al. (2014)
Apoptosis	Mice	H9c2	RES’s ability to prevent DOX-induced cardiomyocyte death is linked to SIRT1-mediated upregulation of p53 deacetylation.	Zhang et al. (2011)
Autophagy	Rats	H9c2	ZLN005 enhances SIRT1 expression and autophagy effectively prevent cardiomyocyte damage caused by excessive hyperglycemia.	Li et al. (2016a)
Oxidative and hypertrophic stress	Mice	HL-1	The *mIGF-1*, through SIRT1 activity, may serve as a promising cardiac therapeutic for protecting cardiomyocytes from oxidative and hypertrophic stresses.	Vinciguerra et al. (2009)
Anoxia/reoxygenation (A/R)-induced injury	Rats	H9c2	A molecule called kaempferol (Kae) is essential for shielding cardiomyocytes from A/R damage via the mitochondrial route, which is mediated by SIRT1.	Guo et al. (2015b)
Myocardial infarction	Mice	HL-1	SIRT1 antisense lncRNA regulates cardiomyocyte proliferation and cardiac regeneration by interacting with Sirt1 mRNA, potentially preventing myocardial infarction.	Li et al. (2018a)
Histone H2AX	Mice	H9c2	SIRT1 controls the phosphorylation of H2AX in cardiomyocytes by deacetylating it, hence preventing doxorubicin-induced cardiotoxicity.	Kuno et al. (2022)
Oxidative, pyroptosis, and apoptosis stress	Mice	H9c2	Activating the *SIRT1/Nrf2* pathway helps melatonin lower oxidative stress, pyroptosis, and apoptosis in Dox-induced cardiomyopathy.	Zhang et al. (2023)
Metabolism and mitochondrial biosynthesis	Mice	H9c2	As a SIRT1 activator, ginsenoside Rc works as a protective mechanism by enhancing energy metabolism and improving cardio- and neuroprotective capacities in both normal and ischemia/reperfusion damage scenarios.	Huang et al. (2021)
IsoRN-induced Injury	Mice	H9c2	Numerous factors affect the IsoRN’s ability to protect cardiomyocytes from damage caused by A/R.	Huang et al. (2016)
Apoptosis	Mice	H9c2	It has been discovered that RES activates SIRT1 to shield H9c2 cells from damage caused by DOX.	Liu et al. (2016b)

### 3.1 SIRT1 and endothelial cells

Endothelial cells play a vital role in the heart and vasculature, serving as a vital conduit between the immunological and circulatory systems. Recent studies explored the pivotal role of SIRT1 in mice lacking CR6-interacting factor (CRIF1), SIRT1 in mitochondrial dysfunction, and its potential as a treatment approach for endothelial dysfunction. The CRIF1 protein is essential for oxidative phosphorylation and peptide synthesis. The study was carried out in a mice model (Shin et al., 2013); CRIF1 deletion causes cardiomyopathy, heightened oxidative stress, inflammation, and malfunction of the heart and mitochondria.

All of these issues resulted from endothelial cells’ reduced production of SIRT1 after the deletion of the CRIFI protein. SRT1720 (SIRT1activator) were injected (intraperitoneally) in knockout CRIFI mice, increasing the endothelial eNOS and decreasing oxidative stress and inflammation. This improved heart function and improved decreased zonula occludens-1 (endothelial junction associated protein) (Piao et al., 2021). In addition, it has been found that SIRT1 plays a crucial role in acute cardiac hypotrophy (AMI) by reducing cardiomyocyte apoptosis and MI size and improving cardiac dysfunction. In an animal model with ligation of the LAD coronary artery in the overexpression and control of SIRT1, lentivirus was administrated to the peri-infarcted region. In primary cardiomyocytes (in post-myocardial infarction), the expression of SIRT1 and Phd3 were reduced, while cleaved caspase-3 and Hif-1 α expression increased. Moreover, in hypoxic conditions, the levels of nuclear SIRT1 and cytoplasmic Phd3 decreased, while cleaved caspase-3 and Hif-1 α expression increased, leads to alleviated hypoxia-induced cardiomyocytes apoptosis (Chen et al., 2025).

**FIGURE 3:**
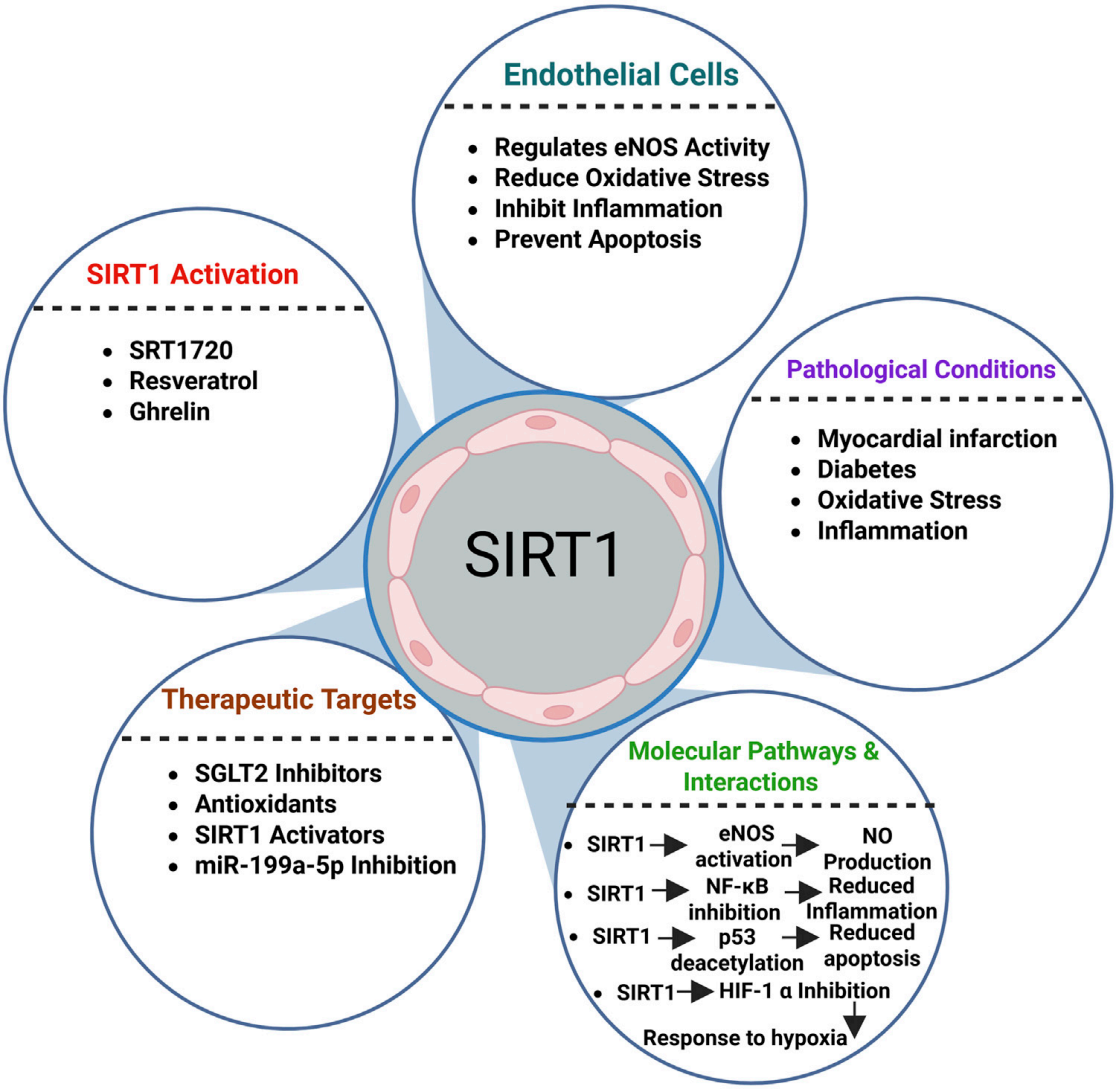
Overview of SIRT1 regulatory functions in endothelial cells (Zhang et al., 2017).

Additionally, SIRT1 which is abundantly expressed in EC, regulates the angiogenic potential and preserves normal endothelial function (Borradaile and Pickering, 2009; Sanchez-Fidalgo et al., 2012). SIRT1 move among the cytoplasm and nucleus in pathological circumstances to alter a number of molecular signaling pathways that help to shield EC from oxidative damage (Hou et al., 2010). Through deacetylating the tumor suppressor p53, SIRT1 specifically inhibits H_2_O_2_-induced premature senescence of EC, while it shields blood vessels from hyperglycemia-induced EC dysfunction via down-regulating p66Shc expression and transcriptionally regulating eNOS (Figure 3) [5]. Additionally, through raising its acetylation, the reduced SIRT1 expression in human EC under high-glucose therapy also causes p53 to become activated (Zhou et al., 2011). A number of substances, including resveratrol (Milne et al., 2007; Orimo et al., 2009), ghrelin (Sanchez-Fidalgo et al., 2012), and Rb1 (Togliatto et al., 2015), have been shown to restore SIRT1 activity, specifically in ob/ob mice exposed to hind limb ischemia. Unacylated ghrelin, the most prevalent form of circulating ghrelin, further protects endothelial cells (EC) from ROS imbalance by reducing their in vivo senescence via SIRT1-mediated p53 and H3K56Ac deacetylation (Song et al., 2014). The protective effect of paeonol, a phenolic compound extracted from cortex moutan (tree peony bark), against H_2_O_2_-induced premature senescence in human umbilical endothelial cells is molecularly elucidated by a reduction in H_2_O_2_-induced upregulation of H3K14Ac and H4K16Ac, which subsequently enhances p53 proliferation through downregulation (Jamal et al., 2014). Additionally, as the development of senescence occurs, SIRT1 mRNA and protein levels gradually decrease because of oxidative stress, which is linked to CVD risk factors. It has been shown that both these stressors induced senescence in mice and that EC senescence was inhibited by SIRT1 overexpression *in vitro* studies. Meanwhile, as a stress response, deacetylation of serine/threonine kinase B1 (LKB1) is consistently elevated (Zhou et al., 2011). Angiogenesis and endothelium-dependent vasorelaxation were all compromised in a mouse model of vascular senescence that was produced through genetically deleting exon 4 of SIRT1 in EC (SIRT1-endo-/-) (Vasko et al., 2014).

Furthermore, it has been established that Yin-yang 1 (Yy1) transcription factor is linked to the expression of circulating soluble ST2 isoform (sST2) in infarcted myocardium. This suggests that elevated sST2 levels are associated with a lower pre-release LV ejection fraction and adverse cardiovascular outcomes. The growth of miR-199a-5p during MI enhances ventricular hypertrophy by enriching the amounts of circulating soluble (sST2), whereas the Yy1/sST2 axis was used for biomechanical stretching. SIRT1 expression is increased and P300 protein is rendered inactive by antimiR199a treatment, resulting in Yy1 inhibition as well as reduced sST2 expression in cardiomyocytes. This significantly reversed the cardiac hypertrophy in HF mice (Asensio-Lopez et al., 2021). Further, MiR-323-3p, a gene highly expressed in rat models and CHD patients, is involved in the progression of the disease. Its expression can be downregulated by SRT1, and over-expression or inhibition of it can lead to increased VEC apoptosis, elevated ac-p65 protein expression, and overexpression of proteins involved in the NF-κB signalling pathway (Du et al., 2021). The function of SIRT1 with long-term administration of Mito-Esc to Apoe-/-mice was investigated; its activation increased the levels of human telomerase reverse transcriptase (hTERT), which postponed senescence of endothelial cells (Karnewar et al., 2022). In transcription inhibition, FOXO6 triggered cardiac microvascular endothelial barrier disruption in the OGD/R model in HCMECs. By inhibiting CTRP3 transcription, FOXO6 causes OGD/R, this then triggers *SIRT1/Nrf2* signalling to tear down the heart microvascular endothelium barrier (Zheng S. et al., 2024).

Furthermore, SIRT1 play a crucial role as a therapeutic target for CVDs. For example, chronic rmSIRT1 supplementation improves endothelial function and vascular compliance in diabetic conditions by enhancing eNOS activity and suppressing NOX-related oxidative stress. This potentially prevents diabetic vascular disease (Yang K. et al., 2023). High glucose levels in CMEC increased ROS, malondialdehyde, and apoptotic cell percentage, it is reversed via the administration of RSV through AMPK/SIRT1 activation (Li et al., 2023). Endothelial dysfunction is prevented by dapagliflozin A (SGLT2) inhibitors via SIRT1 activation, and it is ameliorated by restoring eNOS activity, NO bioavailability, and decreased ROS production (Zhou Y. et al., 2023). In HF mice without diabetes, dapagliflozin, an inhibitor of sodium-glucose-linked transporter 2, decreased EndMT brought on by ISO via deacetylating and breaking down NICD by SIRT1 (Wang W. et al., 2023). DAPA has been shown to activate the *SIRT1/PGC-1α* pathway, which improves endothelial cell mitochondrial function in obese mice (He et al., 2022a). Moreover, homocysteine (a risk factor for CVDs) suppressed STS (a hydrophilic derivative of tanshinone IIA) by activating signalling pathways *SIRT1/NRF2/HO-1*, *NNMT/MMA*, and *AKT/MAPKs* (Zhou Z.-Y. et al., 2023). SIRT1 could potentially promote the inhibition of NLRP3 inflammasome in ECs by L-arginine (semi-essential amino acid) (Zhang M. et al., 2021). Lycopene mitigates oxidative damage in human VECs via activation of the SIRT1/Nrf2/HO-1 pathway, thereby reducing oxidative stress, ROS, cell adhesion, inflammation, and apoptosis (Guo et al., 2023). A trace element called selenium protects against cardiovascular damage by controlling the *SIRT1/p53* and *Cyt-c/Cas-3* pathways (Ilhan et al., 2023). Naringenin (Nar), a bioactive flavanone compound, activates the *AMPKα/SIRT1* signalling pathway, restoring mitochondrial Ca^2+^ and decreasing ROS production as well as upregulating eNOSactivity, increasing NO production, and improving ED (Li et al., 2021). Moreover, melatonin (hormone) regulates the *AMPK/SIRT1* pathway, thus attenuating HG-induced CMEC STZ-induced cardiac dysfunction, oxidative stress, and apoptosis injury (Wang B. et al., 2021). One important treatment target for Kawasaki disease (KD) is the *SIRT1/NF-κB/p65* pathway, which is linked to cardiac dysfunction as well as inflammatory injury to myocardial cells and HCAECs by inhibition of pyroptosis (Yang Y. et al., 2024).

### 3.2 SIRT1 in atherosclerosis and vascular inflammation

Endothelial dysfunction, shown by decreased endothelium-dependent vasorelaxation, is the initial cause of atherosclerosis (Gimbrone Jr and García-Cardeña, 2016). Enhanced oxidative stress, inflammation, reduced autophagy, and decreased NO generation by eNOS and its signalling pathway are among the established causes of atherosclerosis linked to endothelial dysfunction (Donato et al., 2015). In addition to endothelial failure, atherogenesis, which is linked to inflammation and oxidative stress, includes the development of foam cells in the artery walls and the activation and infiltration of immune cells such as macrophages and monocytes (Moore et al., 2013). Additionally, prior research has shown that autophagy in monocytes as well macrophages is essential in the pathophysiology of atherosclerosis by lowering inflammation and oxidative stress linked to insulin resistance. Both hepatic and adipose tissues contribute directly or indirectly to the pathogenesis of atherosclerosis and vascular aging (Lim and Meigs, 2014). Additionally, SIRT1 expression was reduced in HUVECs stimulated with LPS and ATP. The SIRT1 activator inhibited the expression of monocyte chemotactic protein-1 (MCP-1) and C-reactive protein (CRP), whereas SIRT1 knockdown resulted in notable increases in MCP-1 and CRP levels in HUVECs stimulated with LPS and ATP. SIRT1 deficiency enhanced NLRP3 inflammasome activation and the ensuing caspase-1 cleavage, while the NLRP3 siRNA prevented NLRP3 inflammasome activation in HUVECs treated with both ATP and LPS. The NLRP3 inflammasome significantly decreased MCP-1 and CRP production in HUVECs, and the SIRT1 activator therapy decreased MCP-1 and CRP expression levels in collared arteries and IL1-β blood levels (Li Y. et al., 2016). The interaction between these genes was determined using bioinformatics analysis and the dual luciferase reporter gene experiment. The ac-p65 protein, which is linked to the NF-κB signaling pathway, and *miR-323-3p* were both expressed at higher levels in blood samples from patients with mild VEC or CHD atherosclerosis. Increased VEC apoptosis, ac-p65 protein expression, and levels of NF-κB signalling pathway proteins were the outcomes of overexpressing *mir-323-3p* or suppressing SIRT1 (Kang, 2023). It has been shown that dihydromyricetin (DMY), a flavonoid from *Ampelosis grossedentata*, has potential as a therapeutic treatment for atherosclerosis. Deficient SIRT1 has an anti-atherosclerotic role through M1 polarization and regulates the TIMP3/ADAM17 pathways (Jia et al., 2024). In ApoE-/-mice, naringenin treatment increased dyslipidemia, the formation of atherosclerotic lesions, and vascular senescence. It affected vascular senescence and atherosclerosis by activating SIRT1, deacetylating FOXO3a, and modulating PGC1α (Wang J. et al., 2023).

Additionally, the presence of SIRT1 and HAND2-AS1 (antisense-RNA) in plasma from individuals with atherosclerotic plaques and macrophages was derived from THP-1 stimulated by ox-LDL declination (Ma L. et al., 2022). Steroids are the principal treatment for Lupus nephritis (LN), whereas panax notoginseng saponins (PNS) efficiently ameliorate SR and enhance dyslipidaemia in LN through the regulation of the *SIRT1/PPARγ* signalling pathway (Xu et al., 2025). The *Laminaria japonica polysaccharide* (LJP61A) regulates autophagy behavior by up-regulating SIRT1 and FoxO1 and reducing atherosclerosis in HFD-induced LDLr-/-mice; this effect had an effect on siRNA and FoxO1 inhibitors (Li X.-Y. et al., 2022). Additionally, CTRP9, a protein present in the peripheral blood of healthy donors, is essential for lowering cell viability, inhibiting autophagy, and preventing lipid buildup brought on by ox-LDL. It maintains SIRT1 protein levels, upregulates USP22, promotes autophagy and reduces lipid accumulation, leading to a protective effect against atherosclerosis progression (Zeng et al., 2024). The La Ribonucleoprotein Domain Family Member 7 (Larp7), a protein that promotes senescence by decreasing SIRT1 function, led to the development of atherosclerosis in ApoE-deficient mice when subjected to a high-fat diet. Overexpression reduces p16 positive senescent cells in aortic lesions, decreases pro-inflammatory macrophages and SASP factors, and reduces atherosclerotic lesions in HFD-fed ApoEKO (Larp7tetO) mice to prevent atherosclerosis (Yang P. et al., 2024).

### 3.3 SIRT1 and oxidative stress

In addition to atherosclerosis, diabetes, heart failure, age, hypertension, and other conditions affecting the vascular system, oxidative stress is a major cause of CVDs. The accumulation of ROS, which regulates cellular processes, is linked to negative outcomes in CVD. ROS include free radical species, lipid radicals, superoxide anion, nitric oxide, and chemical species with high oxidizing potential. Further, Geniposide (GE), an iridoid glycoside from *Gardenia Jaminoides*, was found to promote Nrf2 transcriptional activation in HFpEF mice, alleviate oxidative stress in H9c2 and HL-1 cells, and reduce oxidative damage brought on by H2O2 using the *MMP2/SIRT1/GSK3β* pathway (Han Y.-L. et al., 2024). In Oa-treated VSMCs, SRT120 reduced mtROS, increased SIRT1 and PGC-1α deacetylation, decreased mtDNA damage, and sped up mitochondrial repair in OA-induced dysfunction (Sung et al., 2024). A member of the CTRP family, *C1q/TNF*-related protein 6 (CTRP6), has been shown to reduce CTRP6 expression in the plasma of heart failure patients’. Its *AMPK/SIRT1/PGC-1α* signaling pathway activation restored mitochondrial homeostasis, which slows the development of heart failure by raising ATP levels and lowering ROS levels (Fan et al., 2024).

Moreover, the overexpression of miR-135 in human and mice cardiac fibroblasts enhances oxidative stress, proliferation, and fibrosis, while inhibiting mitochondrial activity and mediating mitochondrial oxidative respiratory function via SIRT1 to modulate atrial fibrosis (Ding et al., 2024). ACE2 has shown to be important in the development of sepsis-induced cardiomyopathy (SIC) in *C57BL/6* mice. Treatment options include ACE2 inhibitor *MLN-4760* and its activator diminazene aceturate (DIZE); DIZE improved mortality, cardiomyocyte apoptosis, oxidative stress, inflammatory response, and cardiac dysfunction by encouraging MasR-SIRT1-mediated mitochondrial biogenesis, whereas ACE2 inhibitor MLN-4760 exacerbated SIC by preventing MasR-SIRT1-mediated mitochondrial biogenesis (Wan et al., 2024). For ischemic stroke patients, the *SIRT1-BMAL1* pathway is crucial for controlling oxidative stress, according to the expression levels and affecting factors of these pathways. Moreover, ischemic stroke patients have higher levels of oxidative stress and inflammatory markers, such as IL-6, TNF-α, MDA, and SOD, than non-stroke patients. Certain subgroups of ischemic stroke patients had the lowest expression levels of SOD, BMAL1, and SIRT1, whereas other subgroups had the highest levels of MDA, IL-6, and TNF-α (Shi J. et al., 2024). When pathological cardiac hypertrophy in mice was studied, the function of mitochondrial protein homeostasis was examined. Transverse aortic constriction (TAC) was shown to produce less hypertrophy, mitochondrial dysfunction, and oxidative stress damage when C1q-tumor necrosis factor-related protein-3 (CTRP3) was overexpressed. The regulatory protein ATF5 is essential for UPRmt, while SIRT1 was identified as a possible downstream effector molecule. Additionally, during TAC, overexpression of CTRP3-activated UPRmt reduces hypertrophy. By signaling UPRmt via the *SIRT1/ATF5 axis*, CTRP3 decreases oxidative stress damage and mitochondrial dysfunction (Shi L. et al., 2024).

### 3.4 SIRT1 in heart aging and cellular senescence

Extrinsic and intrinsic variables collaborate in influencing the aging rate and aging phenotype. Decline in organ function and heightened disease susceptibility associated with aging underscores the therapeutic importance of identifying the mechanisms governing this process. It has been shown that moderate overexpression of SIRT1 decreased age-dependent apoptosis/fibrosis, senescence markers, cardiac hypertrophy, and cardiac dysfunction. In contrast, high levels of SIRT1 enhanced hypertrophy and apoptosis, thereby causing cardiomyopathy (Finkel, 2005; Alcendor et al., 2007). Cellular senescence, known as the permanent cell cycle, slows down and can be triggered by stresses like aging, ROS, and DNA damage, while in several cardiac types senescence is linked to numerous CVDs, including atherosclerosis, cardiomyopathies, valvular disease of the heart, and arrhythmias (Chen M. S. et al., 2022).

Moreover, Mito-Esc plays a key role in aging mice; its treatment prevents lipid profile formation and improves blood pressure as well as atherosclerotic plaque formation. Activating SIRT1 also raises the amount of human telomerase reverse transcriptase, delaying the senescence of endothelial cells (Karnewar et al., 2022). It has been shown that Metoprolol (β1 receptor blocker) plays a key role in aging; cardiomyocytes are shielded against cellular senescence caused by arginine vasopressin (AVP). It was found to be effective in reducing DNA oxidation, decreasing senescence-associated β-Gal positive cells, and improving telomerase activity. Furthermore, in cardiomyocytes, metoprolol decreases SIRT1 activity, intracellular NAMPT activity, and the NAD^+^/NADPH ratio (Li Q. et al., 2022). SIRT1 is crucial for preventing damage to myocardial contractility since its deletion in young mice’s hearts results in decreased cardiomyocyte contractility and aging-like cardiac dysfunction (Zhang J. et al., 2021). Hydrogen sulphide (H_2_S) has been shown to control pathophysiological processes in the body, such as aging, which is linked to changes in the heart’s structure and function. In the cardiac tissues of elderly rats and cultured aged cardiomyocytes, exogenous H_2_S upregulates the SIRT1-PINK1-parkin pathway, preventing cell death, mitochondrial damage, and oxidative stress while promoting mitophagy (Hao et al., 2022). A novel adipokine known as Isthmin-1 (ISM1) plays a crucial role in aging-related cardiac dysfunction; overexpression of the ISM1 in aging mice mitigates insulin resistance via promoting glucose uptake. ISM1 is essential for reducing myocardial inflammation, cellular aging, and dysfunction in natural as well as in accelerated cardiac aging. It also promotes glycolysis and activates SIRT1 via increasing glucose uptake, this leads to increased SIRT1 activity by O-GlcNAc modification (Hu et al., 2024). In addition, a novel DPP-4 inhibitor anagliptin, was investigated as a potential therapeutic in atherosclerosis, and its treatment improved *TNF- α*, *IL6*, and MCP-1 secretion in VSMC, reduced telomerase activity, and reversed upregulated SIRT1 in IL-1 β treated cells. SIRT1 suppression, however, eliminated the VSMCs’ defense against cellular senescence (Zhao et al., 2021).

Additionally, via phosphorylating the SMAD complex, ST2 enhanced TGFβ signaling *in vitro*, which in turn activated mouse cardiac fibroblasts (MCFs) and suppressed cellular senescence via the signaling pathway of SIRT1/p53/p21 (Tan et al., 2023). ANE can delay cell senescence by inhibiting PARP1 activity, thereby encouraging SIRT1 activity and increasing NAD^+^ release. This approach is a useful anti-aging tactic (Zhao L. et al., 2024). Research has shown that the natural flavone acacetin counteracts the effects of D-galactose-induced myocardial senescence in C57/BL6 mice and H9c2 rat cardiac cells. Oral acacetin administration also improves heart function in animals with accelerated aging by retaining mitochondrial function and boosting mitophagy (Hong et al., 2021). Moreover, doxorubicin-induced subacute senescent cardiomyocytes showed increased expression of markers *P21*, *SA-β-gal*, *Suv39h1* (histone lysine methyltransferase), and *H3K9Me3*, suggesting that downregulation of Suv39h1 reversed the decline of mitochondrial membrane potential. The positive rate of SA-β-gal was lower in the sh-Suv39h1 group, leading to cell senescence inhibition (Yang and Liu, 2024). Endothelial progenitor cells (EPCs) undergo senescence due to TMAO, a process that is associated with age-related diseases. When EPCs were treated with TMAO, Lyc, siAMPK, and siSIRT1, the protective effect was reduced. Lyc, in particular, reduces TMAO-induced EPC senescence via the *AMPK/SIRT1* pathway (Liu et al., 2025). A GLP-1 receptor agonist called ligarglutide (LIR) controls blood sugar levels and reduces vascular stress to help manage diabetes, while also preventing diabetic CVDs by targeting high-glucose induced endothelial cell senescence via the SIRT1-p53/65 signaling pathway (Zhong et al., 2025).

SIRT1 has been shown to control fat accumulation by blocking PPARγ (Picard et al., 2004), enhancing insulin sensitivity, lowering inflammation (Yoshizaki et al., 2010), and restricting preadipocyte hyperplasia via C-Myc deacetylation (Abdesselem et al., 2016). There are SIRT1-dependent biochemical pathways linked to obesity and adipogenesis. For example, nicotinamide mononucleotide adenylytransferases (NMNATs) are enzymes that convert NMN into NAD+, which is required for SIRT1 activity. Notably, lower PARP-1 activity during the early phases of differentiation led to a de-repression of CEBPβ activity, which stemmed from reduced nuclear NAD^+^ levels (Luo X. et al., 2017). Studies have looked into NAD^+^ detection probes that target particular cellular compartments and discovered that cytoplasmic NAD^+^ production from NMN was enhanced by cytosolic NMNAT-NMAT2 induction. This initiated a sequence of events that facilitated 3T3-L1 differentiation by lowering nuclear NAD^+^ levels, inhibiting PARP-1 activity, and de-repressing CEBPβ (Ryu et al., 2018). Adipocyte respiration as well as mitochondrial biogenesis depends on SIR1; it affects adipocytes’ SREBF1c and PPARα signaling as well. NMNAT2 expression is likewise stimulated by NMN via SIRT1 during 3T3L-1 development, but it may be inhibited. Leptin, SIRT1, and PGC-1α expression levels increase when NAD^+^ booster NMN is added to preadipocytes during differentiation, although pro-fibrotic collagen levels decrease (Majeed et al., 2021).

### 3.5 SIRT1 in metabolism and its connection to CVDs

Cardiometabolic disorders, including obesity and T2D, are characterized by the dysregulated systemic as well as organ-specific metabolic profiles and linked to elevated cardiovascular risk (Chen Z. et al., 2022; Costantino et al., 2023). These disorders elevate mortality and morbidity rates, while also straining resources and healthcare systems. The metabolic cardiomyopathy MCM is an important condition within the array of cardiometabolic problems, due to its correlation with the onset of heart failure and mortality (Nishida and Otsu, 2017). MCM is characterized via cardiac hypertrophy and dysfunctional remodeling, occurring without coronary artery disease or hypertension, while a notable aspect of MCM is the altered lipid singling that promotes fatty acid absorption, leading to intramyocardial lipid buildup and lipotoxic injury (Costantino et al., 2019; Suffee et al., 2022). FA-induced lipotoxicity adversely affects cardiomyocytes, inducing apoptosis and elevating stiffness, which leads to a reduction in contractile function (Karbasforooshan and Karimi, 2017). These events are essential for the initiation as well as advancement of MCM and predispose individuals to structural and functional alterations that culminate in HFpEF (Jia et al., 2018; Tan et al., 2020). The db/db mice were compared to their db/+ heterozygous littermates; the mice were given intraperitoneal rSIRT1 for weeks. Metabolic cardiomyopathy (MCM) is a disease in which fat buildup in the heart results in cardiac failure with intact ejection fraction. The H9c2 cardiomyocytes exposed to hyperglycemia served as a model of MCM *in vitro*, and cardiac ultrasonography was utilized to evaluate cardiac function and obtain left ventricular samples. Through improving diastolic performance, fractional shortening, and left ventricular ejection fraction, the rsirt1 therapy maintained cardiac performance and restored cardiac SIRT1 levels. Additionally, it altered the cardiac lipidome by inhibiting the expression of genes linked to lipid transport, metabolism, and inflammation.

PPARG-related genes and intramyocardial triacylglycerols were shown to be greater in people with lower cardiac expression levels of SIRT1 (Majeed et al., 2021). It has been shown that ginsenoside Rc, a substance that targets the SIRT1 signaling system, enhances mitochondrial biogenesis and glucose aerobic metabolism, which increases resistance to cardiac and neurological damage. It was verified that Rc’s interaction with SIRT1’s residue sites promoted its activation. Ginsenoside Rc enhances mitochondrial biogenesis, increases the amounts of complex II-IV of the electron transport chain in neurons and cardiomyocytes, and raises the levels of mitochondrial pyruvate carrier I/II, ATP synthesis, glucose absorption, and hexokinase I/II. Additionally, by reducing PGC1α acetylation through SIRT1 restoration, it activates the PGC1α pathway to induce mitochondrial biosynthesis and decreases mitochondrial damage and apoptosis. This results in SIRT1 activation, which enhances energy metabolism and enhances cardiovascular and neuroprotective processes in both healthy and injured individuals (Huang et al., 2021).

Quercetin pretreatment can reduce ROS and oxidative stress damage in human cardiomyocytes, increase mitophagy, and regulate TMBIM6 expression and endoplasmic reticulum stress, however, transfection with SIRT1 counteracts these protective effects. Quercetin is expected to prevent oxidative stress damage brought on by H/R and control mitophagy and ER stress via *SIRT1/TMBIM6* (Chang et al., 2021a). Another study examined the vascular endothelium in obese mice and HUVECs *in vitro* as a result of sodium-dependent glucose transporters 2 inhibitor DAPA. DAPA reversed the effects of vascular endothelial damage in obese mice and reduced the effects of palmitic acid (PA) on angiogenesis and apoptosis in HUVECs. In addition to enhancing mitochondrial membrane potential, viability, energy metabolism, and biogenesis and improving the structural damage brought on by PA, it also triggered the *SIRT1/PGC-1α* signaling pathway (He et al., 2022a). Another study showed that hyperglycemia can be averted via DAPA through *AMPK/SIRT1* pathway activation; this potentially could become the treatment target for HG-induced damage to endothelial cells (Faridvand et al., 2022).

### 3.6 SIRT1 in aortic stiffness and hypertension

Atrial stiffness and hypertension are often considered prevalent age-related conditions, since their incidence escalates with advancing age (AlGhatrif et al., 2013; Rosano et al., 2013). Arterial stiffening indicates the progressive fragmentation and depletion of elastin fibers, together with the deposition of more rigid collagen fibers in the media of major arteries (Coutinho et al., 2011; Sun, 2015). It transpires independently of atherosclerosis and serves as an autonomous predictor in cardiovascular outcomes (Kaess et al., 2012). Aortic stiffness elevated the systolic blood pressure and the onset of hypertension, demonstrating the correlation among large-artery stiffness and hypertension development (Sun, 2015). It has been shown that the expression and activity of SIRT1 reduces in aortic endothelial as well as mice smooth muscle cells of KL+/−, indicating that klotho deficiency downregulates SIRT1. Moreover, when administered with a selective SIRT1 activator known as SRT1720, arterial stiffness and hypertension were eliminated via Klotho loss in mice muscle cells KL+/−. SRT1720 reversed the klotho depletion which is correlated with substantial reductions in the activity of AMPKα and eNOS in aortas. Additionally, Klotho deficiency increased NADPH oxidase activity, superoxide production, and collagen expression and accelerated elastin disintegration in aortic media (Gao et al., 2016). In another investigation, Klotho deficiency was shown to worsen diastolic dysfunction, exercise intolerance, and cardiac hypertrophy. While the deficiency also enhanced cardiac capillary densities, these abnormalities were mitigated by sKL therapy. The SIRT1 signaling pathway, via the downstream mechanisms of Klotho, promotes diastolic function. Additionally, the reduced Klotho levels were associated with SIRT1insufficiency, however, in mice’s aged hearts, sKL treatment restored SIRT1 expression and alleviated the activation of DNA damage response system. sKL supplementation, then, may be seen as a potential treatment approach for addressing HFpEF in aged mice (Chen and Sun, 2019).

Moreover, angiotensin II (Ang II) elevated expression of SIRT1 via oxidative stress and growth factor receptor-mediated MAP kinase/Akt signaling pathways. These augmented the production of Giα proteins as well as cell cycle proteins, leading to the hyperproliferation of VSMCs. ANG II influences the control of several physiological activities; this includes the proliferation and hypertrophy of VSMCs via the overexpression of Giα proteins (Hossain et al., 2021). Further, it has been shown that in young, healthy arteries, the expression of circ-SIRT1 was abundantly expressed; while in older arteries and the neointima of mice and humans VSMC cells, its expression was lower. The overexpression of circ-SIRT1 reduced neointimal hyperplasia *in vivo* and postponed Ang II-induced VSMC senescence *in vitro*. While at transcriptional as well as post-translational modulation levels, circ-Sirt1 suppressed p53 activity, and it facilitated the SIRT1-mediated p53 deacetylation and inactivation via interacting and holding p53 to prevent its nuclear translocation (Kong et al., 2021). Another study showed that, when neointimal formation occurred after damage, circ-SIRT1 decreased, as did VSMC cells subjected to platelet-derived growth factor BB (PDGF-BB). The localization of circ-SIRT1 in the cytoplasm of VSMCs, where it interacted with c-Myc, (a protein linked to VSMC proliferation), exerted an inhibitory influence on c-Myc activity (Huang et al., 2022).

## 4 Role of SIRT1 in cardio-renal diseases

**FIGURE 4:**
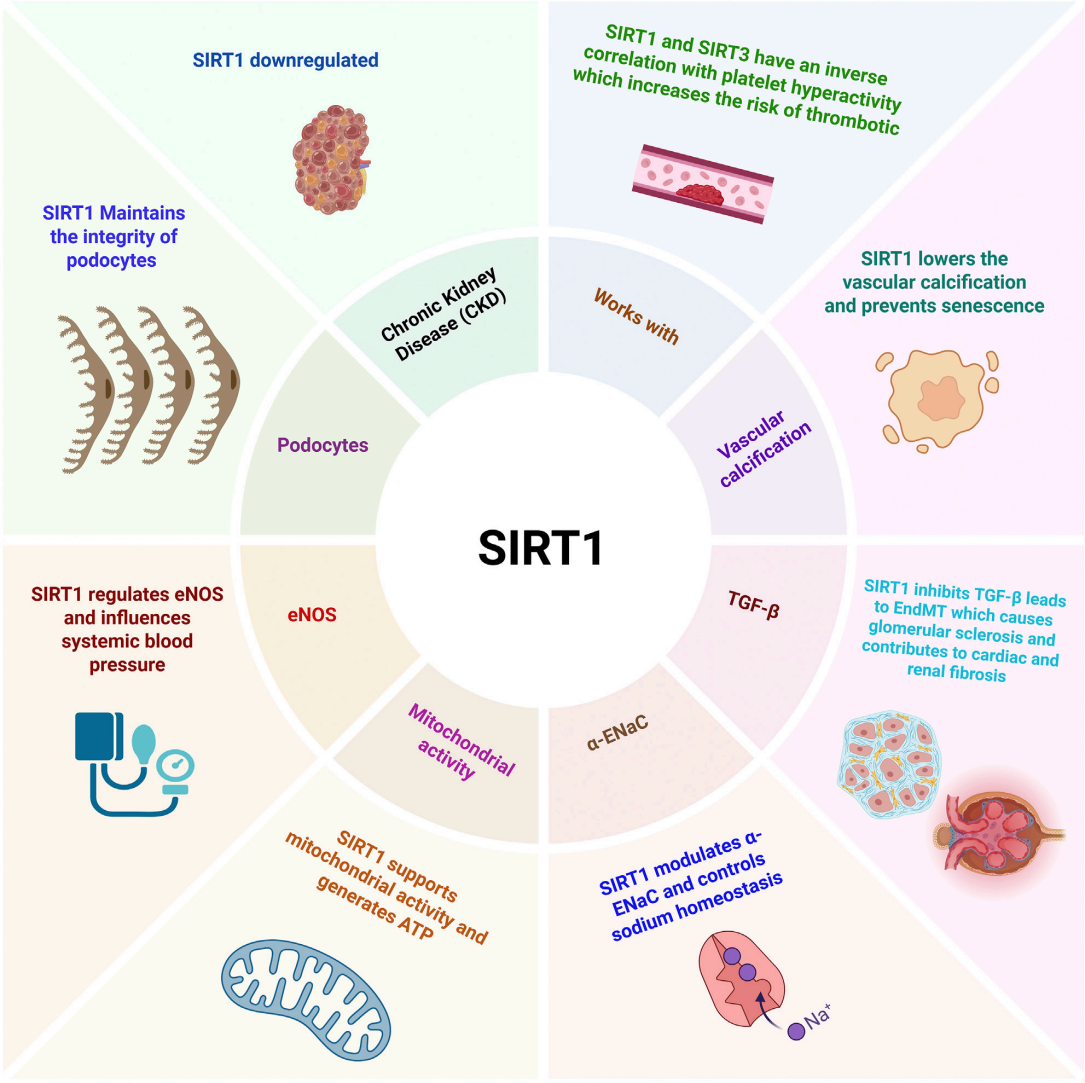
Role of SIRT1 in Cardio-renal diseases: SIRT1 plays a various functional role in CKD, eNOS, vascular calcification, cell senescence prevention, glomerular sclerosis, mitochondrial activity, and sodium homeostasis.

SIRT1 plays an important role in cardio-renal diseases via regulating, inhibiting, and maintaining several pathways (Figure 4). SIRT1 is vital for maintaining the structural and functional integrity of podocytes, which is critical for maintaining the filtration barrier in the kidneys. It not only contributes to podocyte health but also regulates endothelial function by modifying eNOS, thereby influencing systemic blood pressure (Batool et al., 2020). Further, SIRT1 is crucial for mitochondrial activity in the proximal tubule, assuring that tubular cells generate enough ATP to facilitate solute reabsorption (Perico et al., 2021; Pezzotta et al., 2023). At the same time, SIRT1 influences the α-subunit of the epithelial sodium channel (ENaC), in the distal tubules, which is important for controlling water reabsorption and sodium homeostasis (Haschler et al., 2021).

Fibrosis is known to play a key role in the development of heart failure and is also acknowledged as the mechanism that connects the kidney and heart in the development of cardio-renal disorders. Th proliferation of fibroblasts, their differentiation into myofibroblasts, and the consequent deposition of extracellular matrix (ECM) are the causes of fibrosis (Zhang et al., 2009). The endothelial to mesenchymal transition (EndMT) (Zeisberg et al., 2007; Zeisberg et al., 2008), which appears to be a key process leading to glomerular sclerosis in DKD (Kizu et al., 2009), is also a significant element engaged in heart and kidney fibrosis. According to numerous studies, TGF-β is the primary activator and regulator of EndMT as it activates multiple signaling pathways, including TGF/Smad, Erk, and Akt (Pardali et al., 2017). SIRT1 and SIRT3 (both reduced in TGF-β-induced EndMT) appear to be TGF-β inhibiting factors (Li Z. et al., 2018; Lin et al., 2018), so their upregulation may offer a chance to reduce renal and cardiac fibrosis, which would help mitigate cardio-renal syndromes, in which fibrosis is a significant factor (Delgado-Valero et al., 2021). Furthermore, another study showed that, downregulating SIRT1 expression may cause mineral abnormalities in CKD, with the resulting hyperphosphatemia potentially causing a systemic aging phenotype. In vascular smooth muscle cells (VSMCs), SIRT1 activation dramatically decreased phosphate-induced calcification and senescence (Fountoulakis et al., 2025).

Additionally, the novel insights reveal an abnormal secretome in SIRT1-deleted endothelial cells that activates pro-fibrotic tubulointerstitial myofibroblasts and contributes to renal disease, highlighting the significance of distinct paracrine signaling from endothelial cells to adjacent cells and tissues (Lipphardt et al., 2018; Rodriguez and Seta, 2021). Further evidence of cross-talk among various cell types indicates that levels of aortic SIRT1 and SIRT3 inversely correlate with platelet hyperactivity and the subsequent heightened risk of thrombotic events in a model of uremic syndrome. This suggests that dysregulation of vascular SIRT1 and SIRT3 may contribute to an increased risk of atherothrombotic events in patients with chronic kidney disease, a predominant cause of mortality in this demographic (Rodriguez and Seta, 2021).

## 5 SIRT1 preclinical role in cardiovascular diseases

The risk of CVD is almost doubled for patients with hyperlipidemia compared to those with normal total cholesterol levels. Reducing CVDs and averting premature mortality depends on early identification and treatment of hyperlipidemia (Sun et al., 2016). In the heart ventricle, abnormal blood flow and plaque may cause myocardial infarction, which results in congestive heart failure. The most prevalent condition affecting coronary arteries is atherosclerosis, which is characterized by the growth of fibrous tissue in the arterial wall. Furthermore, a number of variables, including inflammation, the associated actions of leukocytes, endothelium, and smooth muscle cells, and the absorption of LDL, are important in the development of atherosclerosis and myocardial infarction. The endothelium of blood arteries is not penetrated by LDL under normal circumstances. Nonetheless, LDL may infiltrate. A number of signaling pathways, such as those connected to the SIRT family, NF-κB, PPAR, and NLRP3, have been linked to inflammation; these pathways can be restored by the right treatment, including statins (Chae and Kwon, 2019). Statins have shown therapeutic benefits in CVDs via the modulation of inflammatory pathways, including eNOS and SIRTs. The use of statins diminishes the expression of the SIRT signaling pathway, resulting in an improvement of CVDs (Sosnowska et al., 2017).

Resveratrol was the first substance to be discovered that mimics the effects of calorie restriction, and it also interferes with more than a hundred other cellular components (Pirola and Fröjdö, 2008). The initial generation of synthetic SIRT1 activating compounds, including SRT1460, SRT1720, and SRT2183, as well as the second generation, which includes SRT2104 and SRT3025, have been developed. These substances successfully activate SIRT1, even though they differ structurally from resveratrol (Camins et al., 2010; Pacholec et al., 2010; Kozako et al., 2022). Considering SIRT1's wide range of functions in many cellular processes and its potential for treating diseases with SRT2104's exceptional efficacy as the most powerful and selective SIRT1 agonist, SRT2104 represents a highly promising therapeutic agent (Chang et al., 2024). Streptozotocin-induced diabetes in mice model (C57BL/6) resulted in decreased SIRT1 protein and inflammation and enhanced aortic contractility and P53 hyperacetylation. SIRT1 protein levels in the aorta of diabetic mice increased 3.79 times after receiving 100 mg/kg of SRT2104 for 24 weeks. Notably, in the diabetic mice discussed earlier, this therapy markedly reduced arterial contraction, inflammation, and oxidative stress, which are all signs of endothelial dysfunction. This indicated that SRT2104 showed remarkable effectiveness in reducing aortic endothelial dysfunction, highlighting its potential as a treatment in diabetic animals (Wu et al., 2018). Another study investigated the role of SRT204 in male diabetic mice, where it enhanced the production of the SIRT1 protein, alleviating ER stress and ameliorating diabetes-induced oxidative damage (Jiao et al., 2018).

In a later clinical study, the effect of SRT2104 on the metabolism of the heart in patients with type 2 diabetes was investigated. For a brief period, patients received 2.0 g/day of SRT2014. Although there were few reported side effects and the chemical was well tolerated, it did not significantly enhance cardiovascular health indicators. This included metrics including cardiovascular efficiency, myocardial energy consumption, and cardiac output (Noh et al., 2017). Another study investigated vascular advantages of SRT2104, specifically its impact on arterial stiffness, a prevalent issue in type 2 diabetes. Patients received short-term treatment and were assessed using recognized clinical parameters. The effect of an oral dosage of 2.0 g of SRT2104 for 28 days on healthy patients’ cardiovascular health was compared with a placebo; every individual showed tolerance, and there were no serious side effects noted (Venkatasubramanian et al., 2016).

Additionally, it has been shown that atorvastatin (5 m/kg/day) administered to Wistar rats for 8 months, decreased MDA levels while simultaneously enhancing the expression of SOD, SIRT1, and eNOS. The expression of SIRT1 correlates with the eNOS ratio and enhances age-related endothelial cell damage (Gong et al., 2014). An *in vivo* study investigated pitavastatin, atorvastatin, and pravastatin at concentrations of 50 and 100 nmol/L, which elevated as a result of Akt phosphorylation. These statins resulted in a decrease of senescence in endothelial cells (Ota et al., 2010). While in endothelial progenitor cells in CAD, atorvastatin and rosuvastatin elevated SIRT1 levels, demonstrating optimal efficacy at dosages of 0.5 as well as 10 μM (Tabuchi et al., 2012). Simvastatin (5 mg/kg/day), exhibited some anti-aging benefits, reducing lipoproteins (LDL and OX-LDL) cholesterol, and, consequently, elevated SRTI expression, which may suppress OX-LDL, thus mitigating vascular endothelial cell damage (Lei et al., 2014). A clinical trial including 108 individuals with a history of premature MI showed that the combination of atorvastatin and simvastatin effectively reduced LDL levels and elevated SIRT1 expression throughout a 3-month treatment duration. Moreover, atorvastatin and simvastatin reduced eNOS levels but did not significantly influence OSI, TAS, or TOS (Yamaç and Kılıç, 2018). Another study investigated the role of atorvastatin (10 mg) and (2.5 mg) administrated to CVD patients, resulting in a decrease in miR-34a levels in the atorvastatin cohort, although no change was seen in the rosuvastatin cohort (Tabuchi et al., 2012).

## 6 Role of other sirtuin family proteins

**FIGURE 5:**
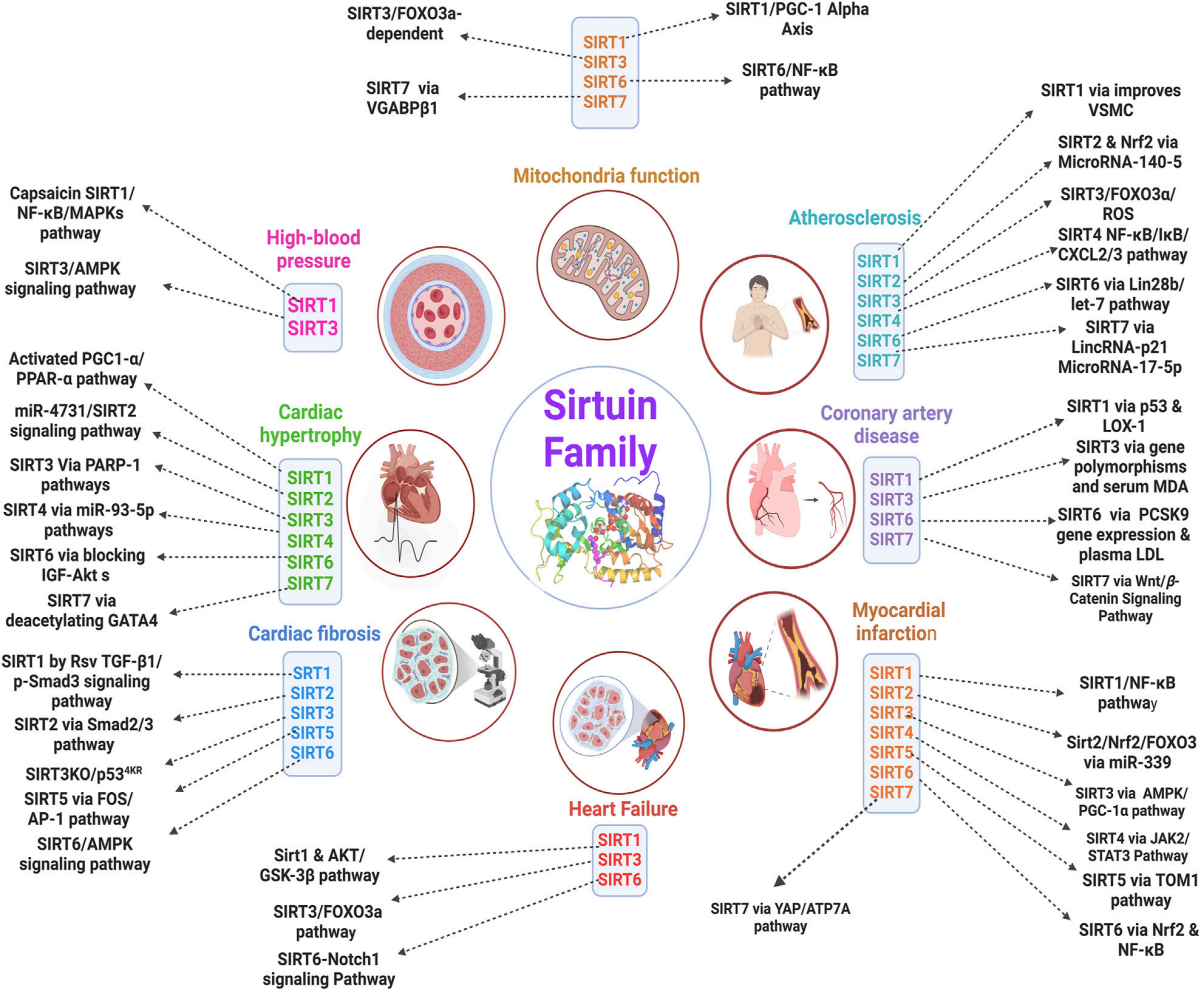
Overview of functional roles and targeted pathways of sirtuin family proteins related to CVDs (Ding et al., 2025).

Histone and nonhistone proteins are deacetylated by a class III deacetylase family called the sirtuin protein family. There are seven members (SIRT 2-7) that are found in the cytoplasm, mitochondria, and nucleus, and they are recognized as mammalian SIR2 orthologs. The sirtuin family plays a role in various CVDs condition by acting on various pathways (Figure 5). Sirtuins are extensively conserved throughout species, ranging from yeasts to primates, and are crucial in connecting aging with diseases. Sirtuins are involved in almost all essential physiological and pathological processes, from embryonic development to stress response and aging. Altered expression and activity of Sirtuins are present in several aging-related disorders, while their activation has shown effectiveness in alleviating certain conditions (e.g., CVDs). This area of study has had rapid and continuous expansion in recent years, including both basic research and clinical trials.

### 6.1 SIRT2 and SIRT3

SIRT2 is essential to biological functions as it acts on the deacetylation of target proteins; its levels reduce with age and various circumstances, resulting in cardiovascular failure. (Table 2; Taneja et al., 2021; Alhasaniah et al., 2024). Sirt2 is essential for AMPK activation, which limits myocardial hypertrophy brought on by age and Ang II. Additionally, it was utilized to target treatment strategies for aging and stress-induced heart hypertrophy (Tang et al., 2017; Lu et al., 2024). EPC-derived exosomal (circ_0018553) reduced Ang II-induced cardiac hypertrophy by effectively regulating the *miR4731/Sirt2* signaling pathway (Zuo et al., 2023). Sirt2, a nuclear protein, modulates the cell cycle by binding and deacetylating FOXO1, which becomes significantly acetylated during autophagy triggered by serum deprivation or oxidative stress. The dissociation of cytosolic *FOXO1-SIRT2* complexes results in heightened acetylation of FOXO (Jiang et al., 2023). The absence of SIRT2 complexes results in heightened acetylation of FOXO (Sola-Sevilla and Puerta, 2024). In the absence of SIRT2, acetylated FOXO1 interacts with *Atg7*, which is essential for starting the autophagic process (Hwang et al., 2023). The studies demonstrates that SIRT2 functions as a unique negative regulator of the NFAT TF, ameliorating heart failure in SIRT2-deficient mice (Bouhamida and Chaudhry, 2023). Furthermore, following an acute cardiac hypotrophy, plasma SIRT2 levels may be a biomarker for heart failure and other serious adverse cardiovascular events (Han R. et al., 2024).

**TABLE 2:** Mechanism of SIRT2 and SIRT3 related to CVDs.

Condition/stress	Model	Cell	Functional role and targeted pathways	References
Oxidative stress and vascular damage	KO mice	HUVECs	SIRT2 and PARP1’s mechanistic relationship in regulating oxidative stress-induced vascular damage.	Zhang et al. (2021c)
MACE and heart failure	AMI (M/F) patients	Plasma	Plasma SIRT2 level predicts heart failure and MACE post-AMI, by regulating metabolic and inflammatory pathways.	Zheng et al. (2021a)
Cardiac hypertrophy and ischemia	Mice (C57BL6J)	Cardiomyocytes	NRF2 is regulated by Sirt2 and, since it stabilizes and increases nuclear translocation, its deletion provides protection.	Yang et al. (2023b)
Coronary heart disease	CHD patients and rat	H9C2	MiR-339, a putative biomarker of CHD, might be treated by down-regulating Sirt2 and using Nrf2/FOXO3.	Shi et al. (2021)
Diabetic cardiomyopathy	Mice (C57BL/6)	AC16, H9c2, and HEK293T	One possible target for DCM therapy and a predictor of DCM is the heart’s SIRT2/CPT2 regulatory axis.	Guo et al. (2025b)
Inflammatory response	Mice (C57BL/6)	Primary neutrophils (Bone marrow)	The inhibitory effect of colchicine on NLRP3 inflammasome activation was reduced when SIRT2 was suppressed.	Sun et al. (2022)
Cardiomyocyte hypertrophy	Human and Mice (C57BL/6)	Cardiomyocytes	PHF19 epigenetically regulated SIRT2, which aided in the development of heart hypertrophy.	Gu et al. (2021)
Diabetic angiopathy	Mice	HUVECs	In SIRT2-mediated NLRP3 deacetylation, 1,8-cineole is essential for anti-pyroptotic and anti-inflammatory processes with diabetic angiopathy.	Zhang et al. (2024a)
Autophagy and acute liver failure	Male mice (C57BL/6) WT	AML12	SIRT2 and AMPK are important for autophagy in acute liver failure.	Zhang et al. (2024c)
Obesity	Male mice (C57BL/6)	3T3-L1	Rhein’s binding to SIRT2 inhibits NLRP3 inflammasome activation in macrophages, promoting white adipose tissue thermogenesis during obesity.	Zhou et al. (2024c)
Coronary atherosclerosis	Patients (T2DM)	HAECs	SIRT3 has been associated with the advancement of atherosclerosis in individuals with type 2 diabetes by raising PPAR-α and eNOS levels while lowering iNOS levels.	Gong et al. (2022)
Diabetic cardiomyopathy	Mice (db)	Cardiomyocyte	Through the restoration of mitochondrial integrity, prevention of cardiomyocyte cell death, and promotion of SIRT3 deSUMOylation via SENP1, BAI improves DCM.	Zhang et al. (2025)
Cardiac fibrosis	Mice	CFs	Heart fibrosis was prevented by QU via the regulation of the *SIRT3/TGF-β/Smad3* signaling pathway.	Lin et al. (2024)
Diabetic cardiomyopathy	Mice (DCM)	NRCMs	Macrod1 is a potential target for DCM treatment by targeting the PARP1-NAD^+^-SIRT3 pathway.	Liu et al. (2024b)
MIRI	Male Mice (C57BL/6)	H9c2	Ramelteon enhances MIRI by stimulating the Sirt3 signaling pathway	Yang et al. (2024c)
Hypertension	Rat (Maternal hypothyroidism)	A7r5	Preventing hypertension and controlling blood pressure depend heavily on the T3-THRA1/PGC-1α/SIRT3 cascade.	Guo et al. (2025a)
Cardiac remodeling	Mice	NRCMs	In order to preserve mitochondrial structure and function and shield the myocardium from cardiac remodeling, SIRT3 controls PPARγ-mediated CL biosynthesis.	Liu et al. (2024a)
Cardiac problems linked to obesity	Male mice (C57BL/6J)	H9c2	EMPA, activated via SIRT3, mitigates cardiac damage in obese mice by promoting autophagosome formation and elongation via AMPK/Beclin1 and ATG4B/ATG5.	Luo et al. (2024)
Myocardial hypertrophy and fibrosis	Rate and (SIRT3 KO mice)	H9c2	2-APQC is a SIRT3 activator that regulates mitochondrial homeostasis, alleviating myocardial hypertrophy and fibrosis.	Peng et al. (2024)
Myocardial ischemic	Male mice (C57BL/6 J)	Heart tissue	By promoting the SIRT3/FOXO3a signaling pathway, resveratrol shields obese animals against the damaging effects of cardiac ischemia.	Zhu et al. (2024)
Mitophagy and apoptosis	Rat (HF)	H9c2	SFYX is known to protect against heart failure by activating SIRT3/FOXO1 signaling-mediated mitophagy and apoptosis through deacetylation.	Li et al. (2025)
Myocardial (I/R)	Rat (I/R)	H9c2	Through the SIRT3-SOD2-mtROS pathway, NR supplementation decreased oxidative stress, which in turn decreased mitochondrial damage and apoptosis after myocardial I/R injury.	Zhao et al. (2024a)
MIRI	Mice (SI/R)	H9C2	One important PTM of SIRT3 during SI/R is O-GlcNAcylation, which offers information on a potential mechanism for future treatment strategies.	Zhou et al. (2024b)
Autophagy	Male mice (C57BL/6 J) WT	Primary cardiomyocyte	Through mechanisms regulated by SIRT3, PARKIN regulates mitophagy.	Lu et al. (2025)
Cardiac hypertrophy	Rats (Dahl/SS) and (SHRs)	HL-1 cardiomyocytes and Primary cardiomyocytes	Canagliflozin SIRT3 activator has therapeutic effects on cardiac hypertrophy.	Zhao et al. (2025)
I/R	Mice (C57BL/6 J) I/R	HL-1	NR alters the SIRT3/mtROS/JNK pathway and raises NAD^+^ levels, which greatly prevents myocardial I/R damage.	Wang et al. (2024a)
SIMI	Male mice (C57BL/6 J)	HL-1 myofibroblasts	ANXA1sp reduced sepsis-induced MI by increasing SIRT3 expression, which promotes mitochondrial biosynthesis and inhibits oxidative stress as well as autophagy.	Qin et al. (2024)

Human SIRT3 (hSIRT3) has two isoforms, and its location is highly debated, localized either in the cytoplasm and nucleus. The longer isoform a full-length protein that is 44 kDa long (Adolph, 2023). When mitochondrial matrix processing peptidase (MPP) cleaves the N-terminal mitochondrial localization sequence (MLS) of SIRT3, a 28 kDa isoform of hSIRT3 is produced. The longer isoform is then processed by proteases into a functionally active mitochondrial deacetylase (He et al., 2024). SIRT3 is an essential mitochondrial enzyme implicated in energy balance, cardiac remodeling, and heart failure. Its expression reduces with aging, cardiovascular conditions, and metabolic disorder (Table 2; Zhang et al., 2024d). SIRT3 protects the heart against hypertrophy, cardiac dysfunction, and stress-induced cellular apoptosis. Clinical investigations indicated that coronary microcirculation abnormalities were seen among individuals with heart failure and a maintained ejection fraction (Liu et al., 2023; Zhang et al., 2024d). According to *in vitro* studies, the expression of SIRT3, PPAR-α, and eNOS proteins was elevated in a concentration-dependent way by high-glycemic (HG) levels (Tsetegho Sokeng, 2019; Liu L. et al., 2021). HAECs had lower levels of iNOS expression, while overexpression of SIRT3 in cells inhibited iNOS expression. SIRT3 is associated with the development of atherosclerosis among individuals with type 2 diabetes via upregulating eNOS and PPAR-α (Gong et al., 2022). In endothelial cells, SIRT3 promotes the anti-inflammatory, anti-autophagic, and antioxidant effects of PCSK9 inhibitors, also decreasing LDL. These inhibitors imply that SIRT3 may be a modulator of this pleiotropy (D'Onofrio et al., 2023). In order to improve vascular resilience and protect the human microcirculation from stress, SIRT3 may be a suitable therapeutic target due to its crucial role in compensatory signaling during flow in human arterioles (Jobe et al., 2024). Through the activation of promoting autophagosome formation, EMPA reduces heart damage in obese mice by promoting autophagosome membrane elongation via *ATG4B/ATG5* and starting autophagy via AMPK/Beclin1 (Luo et al., 2024). When rats were fed high-salt diets, it was shown that hypertension may cause atrial fibrillation (AF) and alteration of lipid metabolism; AF sensitivity was increased when the SIRT3/AMPK signaling pathway was inhibited (Wang X.-H. et al., 2024).

### 6.2 SIRT4 and SIRT5

The heart, kidneys, brain, and liver all have the mitochondrial sirtuin SIRT4, which is an effective ADP-ribosyltransferase on histones and bovine serum albumin but lacks NAD^+^ dependent deacetylase activity (Poulose and Raju, 2015). SIRT4 exerts protective effects via its anti-apoptotic function and regulates insulin synthesis, mitochondrial gene expression, and fatty acid oxidation. SIRT4 serves a contentious function within the cardiovascular system; based on the situation, it can be beneficial or detrimental. In mice given ANG2, it causes hypotrophy, fibrosis, and heart failure (Table 3) and it stimulates mitochondrial fusion in SIRT4-transfected HEK293 and fibroblast cells, inhibits mitophagy through linking to the protein ocular atrophy 1 (OPA1), and increases ROS buildup by connecting with SIRT3, which stops SIRT2 from activating (Han et al., 2019; Eid et al., 2021). ATP synthesis in mammalian cells is linked to SIRT4 expression. For this process, the adenine nucleotide translocase 2 (ANT2) facilitates the entry of ADP into the mitochondria and the exit of ATP. Crucially, ANT2 must be deacetylated for this transport to function effectively. In the cardiovascular system, SIRT4 facilitates mitochondrial fusion in SIRT4-transfected HEK293 cells and fibroblasts, prevents mitophagy by binding to the protein known as ocular atrophy 1 (OPA1), and increases ROS buildup through its relationship with SIRT3, which stops it from activating SOD2 (Zullo et al., 2022). SIRT4 modulates myocardial hypertrophy by influencing transcription levels, while MiR-497 targets SIRT4, and its overexpression can inhibit SIRT4 both *in vitro* and *in vivo* (Zhang and Ni, 2021). Furthermore, by affecting the *miR-93-5p/SIRT4* pathway, the lncRNA MALAT1 may reduce cardiac hypertrophy. MALAT1 also regulates SIRT4 production by degrading *miR-93-5p* (Xu et al., 2023). It has recently been shown that in male mice (C57BL/6 sirt4-Tg) treated with Ang II, the Sirt4-mediated hypertrophic response was inhibited by manganese porphyrin, a SOD mimic (Luo Y.-X. et al., 2017). By raising ROS levels, Sirt4-mediated hypertrophic response was inhibited through the development, fibrosis, and cardiac dysfunction, pointing to a possible involvement for Sirt4 in pathological cardiac hypertrophy (Ramachandra et al., 2021). Additionally, overexpression of SIRT4 reduces myocardial infarct size and serum creatine phosphokinase levels, while siRNA depletion amplifies these parameters. The protective functions of SIRT4 against cardiac ischemia-reperfusion damage are associated with maintained mitochondrial activity and reduced myocardial apoptosis. It may improve myocardial ischemia-reperfusion damage by modulating mitochondrial activity and apoptosis, yielding therapeutic advantage (Christidi and Brunham, 2021). SIRT4 overexpression reduces DIC by improving cardiac function and lowering cardiomyocyte autophagy and apoptosis. It may also provide protection against DIC by inhibiting *Akt/mTOR*-dependent autophagy (He et al., 2022b). The oxLDL administration decreased SIRT4 expression in HUVECs, influencing cell mortality and proliferation, and overexpression of SIRT4 reduced cellular mortality and enhanced proliferation. By obstructing PI3K phosphorylation and p65 NF-κB expression, it blocked the *PI3K/Akt/NF-κB* pathway, enhancing HUVEC viability, diminishing inflammatory cytokine production, and suppressing the signaling pathway (Tao et al., 2019).

**TABLE 3:** Mechanisms of SIRT4 and SIRT5 related to CVDs.

Condition/stress	Model	Cells/tissue	Functional role and targeted pathways	References
Atherosclerosis	Mice	THP-1	Atherosclerosis is caused by Sirt4 deficiency, which stimulates the *NF-κB/IκB/CXCL2/3* pathway.	Chang et al. (2023)
Cardiotoxicity	Mice (C57BL/6) male	Cardiomyocytes	Overexpression of SIRT4 prevents DIC via preventing Akt/mTOR-dependent autophagy.	He et al. (2022b)
Endothelial dysfunction	Human	HUVEC and TeloHAEC	In sepsis, the *miR-15b-5p–SIRT4* axis may be a target for LPS-induced inflammatory pathways, whereas i-PCSK9 guards against vascular injury.	Martino et al. (2023)
Cardiac hypertrophy	Male mice KO &Tg (C57BL/6)	Cardiomyocytes	In response to pathogenic activation, Sirt4 raises ROS levels, which causes hypertrophic growth, the development of fibrosis, and heart failure.	Luo et al. (2017b)
Apoptosis (hypoxia)	Hypoxic (H9c2)	H9c2	SIRT4 is essential for hypoxia-induced cardiomyocyte death and may help cure ischemic hearts.	Liu et al. (2013a)
(MI-R) injury	Mice	Cardiomyocytes	SIRT4 regulates mitochondrial function and apoptosis, potentially enhancing MI-R injury.	Zeng et al. (2018)
Aging	Human and Mice (C57BL/6)	ASM, A549, HMVEC-L, and EC	Role of resveratrol in human ASM cells is determined by the NAMPT-SIRT4-hTERT axis.	Huang et al. (2015)
Cardiac hypertrophy	Mice	NMC	MiR-497, a potential therapeutic substance, has been found to modulate cardiac hypertrophy by targeting Sirt4.	Xiao et al. (2016)
Cardiac hypertrophy	KO Mice (C57BL/6	Cardiomyocytes and fibroblasts	Sirt4 raises ROS levels, which leads to hypertrophic growth and heart malfunction.	Liu et al. (2016a)
Diabetic cardiopathy	Mice (C57BL/6-Sirt5t^m1cyagen^) KO and Diabetic patients	Cardiomyocytes	Sirt5 and CPT2 may be useful targets for DbCM treatment since Sirt5 deficiency inhibits FAO and causes myocardial lipotoxicity in diabetic hearts via Lys424 succinylation in CPT2.	Zheng et al. (2024b)
Metabolism and inflammation	H9c2 (CRL-1446)	H9c2 (CRL-1446)	CAT protects against cardiac damage by controlling inflammation and energy metabolism via the SIRT5-mediated signaling pathway.	Zheng et al. (2024b)
Heart Failure	Mice (C57BL/6J)	HL-1	Through SIRT5, quercetin enhances IDH2 desuccinylation and preserves mitochondrial homeostasis.	Chang et al. (2021b)
Diabetes	Mice (C57BL/6)	HepG2	DMBG increases hepatic glucolipid metabolism by desuccinylation of ECHA via SIRT5.	Tang et al. (2025)
Cardiac pressure overload	Mice (C57BL/6J) KO	Heart tissue	Under conditions of pressure overload, SIRT5 is essential for sustaining heart oxidative metabolism.	Hershberger et al. (2017a)
Mitochondrial dysfunction and cardiac	Rat (SD)	NRCMs	SIRT5 is essential for mitochondrial dysfunction as well as cardiac hypertrophy triggered by RIP140.	Liang et al. (2025)
Diabetic cardiomyopathy	Mice (C57BL/6N) KO	Cardiomyocytes and fibroblasts	SPI1 reduces DCM-associated cardiac damage by transcriptionally activating SIRT5, which mediates GSTP1 Mal-Lys alteration and ensures the stability of proteins.	Wei et al. (2024)
IR injury	Mice (C57BL/6) KO and human heart tissue	HL-1	ANT2 and VDAC1 interact via a Sirt5-regulated pathway to cure cardiac damage and preserve mitochondrial homeostasis.	Li et al. (2024a)
MI	Rat (SD)	AC16 cells (Human)	Desuccinylation of TOM1 by SIRT5 regulates autophagy-induced cell death in MIRI.	Li et al. (2024b)

Metabolic activities such as the Krebs cycle, glycolysis, oxidation, fatty acid, and urea cycle are regulated by mitochondrial SIRT5 (Bringman-Rodenbarger et al., 2018). It has been shown that mice with sirt5 silencing had lower expression levels of the fibrinolysis inhibitor PAI. The absence of Sirt5 in the diabetic heart results in diminished fatty acid oxidation but does not influence fatty acid absorption capacity, leading to the buildup of fatty acid intermediate metabolites such as medium and long-chain fatty acyl-carnitines. The functional succinylated substrate mediator of SIRT5 is recognized to be CPT2, an enzyme that catalyzes the conversion of fatty acyl-carnitines into fatty acyl-CoA. The absence of SIRT5 causes Lys424 in CPT2 to become succinylated, which in turn causes the accumulation of fatty acyl-carnitines and the inactivation of its enzymatic activity. The CPT2 (K424R) mutation mitigated the disruption of fatty acid oxidation and lipid accumulation brought on by SIRT5 deletion, indicating that SIRT5 and CPT2 could be therapeutic targets for diabetic heart disease (Wu et al., 2024). Cardiomyocyte apoptosis brought on by oxidative stress is mostly controlled by SIRT5, and pharmacological treatments that target SIRT5 expression may help repair heart damage brought on by oxidative stress (Liu et al., 2013b). Additionally, it has been shown that quercetin, a component in medicinal plants, provides therapeutic benefits against heart conditions. Quercetin has demonstrated the ability to enhance cardiac function and reduce myocardial fibrosis in animal models of heart failure and myocardial fibrosis by improving mitochondrial energy metabolism and modulating mitochondrial fusion/fission and biosynthesis; however, cell survival was affected as it encouraged SIRT5 expression to desuccinylate IDH2, and si-SIRT5 treatment eliminated quercetin’s protective impact on cellular survival (Chang et al., 2021b). In obese mice, SIRT5 plays a key function in lipid metabolism and the browning of white adipose tissue. The absence of SIRT5 elevates UCPI succinylation, leading to diminished capacity and compromised cold tolerance; the SIRT5-C/EBPβ axis governs energy equilibrium and metabolism associated with obesity (Zhai et al., 2024).

### 6.3 SIRT6 and SIRT7

SIRT6 and SIRT7 significantly influence cardiovascular diseases by regulating several pathways (Table 4). The overexpression of Nrf2 affects the progression of CAD by decreasing NF-kB expression and enhancing the production of antioxidant genes (Divya et al., 2024). NF-kB causes endothelial dysfunction by increasing the production of inflammatory cytokines (Casper, 2023). The Nrf2 pathway can control SIRT6 and NF-κB, preventing CVDs by reducing the generation of ROS and reducing inflammation by downregulating NF-kB transcription (Divya et al., 2024). Overexpression of SIRT6 preserves telomere integrity, delays senescence, and decreases expression of inflammatory cytokines. Endogenous SIRT6 deacetylase plays a key role in VSMC senescence and atherosclerosis (Grootaert et al., 2021). A recent study has shown that, during I/R, SIRT6 is essential for controlling OS and myocardial damage (Liu G. et al., 2021 SIRT6 plays a crucial role in preventing hypertension and its complications. It controls ACE2, possibly by reducing nuclear p-ATF2 accumulation in CC-induced endothelial dysfunction, a known risk factor for hypertension (Zheng Z. et al., 2021). Additionally, SIRT6 maintains endothelial function and prevents hypertension through Nkx3.2-GATA5 signaling (Guo et al., 2019). It was shown that CMECs treated with HG+PA had significantly lower SIRT6 expression, which exacerbated DCM. Sirt6-KOEC exacerbated DCM by decreasing cardiac function and increasing perivascular fibrosis and cardiomyocyte hypertrophy; SIRT6 is linked to EndMT via the *Notch1* signaling pathway (Zhang et al., 2020). According to study findings, cholesterol crystals (CCs) have the ability to decrease eNOS levels, increase adhesion molecules, and endocytose ECs (Jin et al., 2020). They also inhibit the production of SIRT6, which might decrease endothelial dysfunction (He Y. et al., 2021). SIRT6 reduction impairs vascular endothelial function in hyperlipidemic mice, according to *in vivo* investigations (Jin et al., 2020). Increased SIRT6 activity could be a viable treatment approach for atherosclerotic disease as it is essential for preserving endothelial function (Xu et al., 2016). SIRT6 on EndMT in mice and human HUVECs revealed that co-treatment with TNF-α and IL-1β promotes EndMT and reduces SIRT6 expression (Vijakumaran et al., 2023).

**TABLE 4:** Mechanisms of SIRT6 and SIRT7 related to CVDs.

Condition/stress	Model	Cells/tissue	Functional role and targeted pathways	References
Heart failure	Mice (C57BL/6) TAC	Heart tissue	By modifying telomeres, SIRT6 prevents damage to the myocardium.	Li et al. (2017)
I/R injury	Mice (C57BL/6) KO	Cardiomyocytes	SIRT6 protects the heart from I/R injury by activating FoxO3α in the ischemic heart, upregulating antioxidants and suppressing oxidative stress through AMP/ATP-induced AMPK-dependent mechanisms.	Wang et al. (2016)
Diabetic cardiomyopathy	Mice (C57BL/6) db	Rat (H9c2) and heart tissue	By regulating each other’s activity, Sirt6 and Sirt3 shield the heart against diabetes-mediated cardiomyopathy brought on by obesity.	Kanwal et al. (2019)
Endothelial inflammation	Mice (C57BL/6) KO	HUVECs	SIRT6 reduces the inflammatory response of the vascular endothelium, which in turn suppresses EndMT.	Chen et al. (2021)
Aging and cardiac hypertrophy	Mice (C57BL/6) KO/Tg	H9c2	Sirt6 prevents the heart from experiencing age-induced cardiac hypertrophy and fibrosis by controlling a number of cellular processes linked to aging.	Pillai et al. (2021)
Diabetic cardiomyopathy	Mice (C57BL/6) KO	CMECs	In CMECs activated with HG+PA, Sirt6 participates in EndMT via the Notch1 signaling pathway.	Zhang et al. (2020)
Hypertension and cardiorenal	Mice (C57BL/6) KO and Rat	RGECs	SIRT6 keeps the endothelium function intact, preventing hypertension and its consequences.	Guo et al. (2019)
Endothelial dysfunction	Mice (C57BL/6) and Human	HUVECs	SIRT6 regulates ACE2 by blocking the accumulation of nucleus p-ATF2 in CC-induced endothelial dysfunction.	Zheng et al. (2021b)
Arterial thrombosis	Mice (C57BL/6) KO	HAECs	SIRT6 knockdown increases TF expression and initiates pro-inflammatory pathways, protecting endothelium SIRT6 from experimental arterial thrombosis.	Gaul et al. (2023)
Obesity and oxidative stress	Mice (C57BL/6)	Cardiomyocytes	Because SIRT6 regulates ENDOG/SOD2, it helps reduce myocardial oxidative stress caused by obesity resulting from a high-fat diet.	Gao et al. (2023)
Hypertension	Rat	CFs	According to the findings, SIRT7, by controlling YAP/ATP7A signaling, lowers cuproptosis, myocardial fibrosis, and cardiac dysfunction in hypertensive conditions.	Chen et al. (2024)
Pulmonary hypertension	Mice KO and Tg	PAECs and HEK293T	Targeting SIRT7 specifically in the pulmonary endothelium may be a PH treatment approach, as the SIRT7/KLF4 axis maintains PAEC homeostasis.	Zhang et al. (2024b)
Atherosclerosis	AS Mice and human	VSMCs	MiR-17-5p was downregulated by p53-dependent lincRNA-p21 expression, which subsequently prevented AS development by increasing SIRT7.	Wang et al. (2021b)
Endothelial senescence	Human	HUVECs and ECs	MiR-335-5p has been discovered to downregulate SIRT7 expression in human cells, pointing to possible treatment targets for age-related illnesses such ASCVD.	Liu et al. (2022)
Genotoxic stress	Mice C57BL/6 KO	MEFs, U2OS and 293T HEK	Method for p53 tumor suppressor stability in response to genotoxic stress that is reliant on SIRT7.	Ianni et al. (2021)
Angiogenesis	Rat (SD) and MCAO	HUVEC	AST IV targets the SIRT7/VEGFA signaling pathway to promote angiogenesis, which may lessen brain tissue damage after CI.	Ou et al. (2023)
Myocardial tissue reparation	Mice C57BL/6 KO, MI and HI	Tissues	SIRT7 regulates autophagy and plays a crucial role in tissue repair by maintaining TGF-β I.	Araki et al. (2015)
HAVSMCs proliferation and migration	Human	HAVSMCs (T/G)	SIRT7 effectively inhibited the proliferation and migration of HAVSMCs by enhancing Wnt/β-catenin activation.	Zheng et al. (2018)
Cardiac hypertrophy	Mice C57BL/6 KO	NRCMs	Through its interaction and promotion of GATA4’s deacetylation, Sirt7 exerts an antihypertrophic impact.	Yamamura et al. (2020)

Adenovirus-mediated overexpression of SIRT6 inhibits inflammation-induced EndMT, but SIRT6 knockdown enables it and suppresses EndMT by reducing the inflammatory response of vascular endothelial cells (Chen et al., 2021). Moreover, it has been demonstrated that SIRT6 overexpression significantly augmented autophagic flux in macrophages, inhibited apoptosis, and lowered the expression of VCAM-1, ICAM-1, and P-selectin. This in turn reduced macrophage and foam cell infiltration and suggests a novel therapeutic strategy for enhancing atherosclerotic plaque stability (Wang et al., 2020). SIRT6 inhibits the synthesis and activation of NAD(P)H oxidase, which controls vasomotor function in conduit arteries. In animals lacking the SIRT6 haplotype, NAD(P)H oxidase improves endothelial function, activation, and/or histone acetyltransferase inhibition (Greiten et al., 2021). SIRT6 plays a key negative regulator of atherosclerosis progression and endothelial dysfunction (Liu Z. et al., 2016), and the suppression of SIRT6 in vascular smooth muscle cells may lead to vascular calcification via impairing DNA damage repair processes (Wang et al., 2022).

Moreover, it is believed that the SIRT7 mutant mice’s cardiac condition is partially caused by increased p53 activation due to a lack of SIRT7-mediated deacetylation (Vakhrusheva et al., 2008). It was shown that mice receiving high doses of vitamin D had hardened arteries with reduced SIRT7 expression. The *SIRT7/KLF4* axis modulates pulmonary artery endothelial cell homeostasis by influencing proliferation, migration, and tube formation. PAEC dysfunction may be ameliorated either through NAD^+^ supplementation or overexpression of SIRT7 driven by an adeno-associated virus type 1 vector, leading to favorable pulmonary hypertension phenotypes (Zhang et al., 2024b). Apoptotic processes and mitochondrial dysfunction in human aortic endothelial cells (teloHAECs) exposed to IL-6 were examined in relation to *miR-148a-3p*. The expression of miR-148a-3p reduces during IL6-activated inflammatory pathways, opposing cytokine production and apoptotic cell death and, in turn, enhancing mitochondrial redox homeostasis and respiration (Anastasio et al., 2024).

## 7 Future prospective and conclusion

Current research has developed the key role of SIRT1 signaling in preventing CVDs. Recent investigations focus on the therapeutic potential of SIRT1 for atherosclerosis, vascular aging, inflammation, and glucose metabolism disorder. Understanding of SIRT1 signaling in CVD protection remains critical. The cellular redox state modulates SIRT1 activity, enabling antioxidants to act by key pathways (*SIRT1/FOXOs*, *SIRT1/NF-κB*, and *SIRT1/p66Shc*) to protect CVD Further complexity arises from the regulatory crosstalk involving transcription factors (TFs) such as NF-κB and sirtuins. This intricate relationship is further evidenced by the overlap of regulatory pathways with microRNAs (e.g., miR-34a), collectively highlighting the interconnected nature of sirtuin-mediated protection. Emerging research implicates SIRT1 as a key regulator of inflammatory pathways in CVDs, especially through its modulation of *-κB/NKG2D* signaling in atherosclerotic plaque formation. Notably, intracellular glutathione levels during oxidative stress appear critically important, as they directly influence SIRT1 activity and its downstream effects.

Although SIRT1 enhances genomic stability and longevity, making it an attractive target related to CVDs modulation, comprehensive understanding of its molecular targets is still required. This review highlights SIRT1 signaling in CVDs protection, emphasizing its shared molecular targets as key therapeutic candidates. Major challenges in the field include elucidating the SIRT1 signaling cascade as well as understanding the role of SIRT1 in redox regulation during vascular aging and pathogenesis of CVD. Future research should focus on mapping the human disease epigenomic to identify stage-specific targets. Moreover, lifestyle interventions such as caloric restriction and exercise mitigate CVD-related oxidative stress, and the long-term effects of pharmacological SIRT1 modulation requires careful evaluation.
